# The Associations Between the TyG Index and the Risk of Cancer—A Systematic Review and Meta‐Analysis

**DOI:** 10.1002/cam4.71232

**Published:** 2025-10-02

**Authors:** Hongyu Li, Zijie Chen, Guoheng Jiang, Wenqian Yu, Jing Luo, Shiyi Li, Linjun Xie, Xuan Bai, Yiting Xu, Yi Jiang, Menglin He, Min Mao, Xin Wang

**Affiliations:** ^1^ Department of Epidemiology and Biostatistics, West China School of Public Health and West China Fourth Hospital Sichuan University Chengdu China; ^2^ Health Promotion and Food Nutrition & Safety Key Laboratory of Sichuan Province West China School of Public Health and West China Fourth Hospital, Sichuan University Chengdu China; ^3^ Department of Pediatric Pulmonology and Immunology West China Second University Hospital, Sichuan University Chengdu China; ^4^ Key Laboratory of Birth Defects and Related Diseases of Women and Children (Sichuan University), Ministry of Education Chengdu China; ^5^ Sichuan Birth Defects Clinical Research Center, West China Second University Hospital Sichuan University Chengdu China

**Keywords:** cancer, insulin resistance, meta‐analysis, systematic review, triglyceride‐glucose index

## Abstract

**Background:**

The triglyceride glucose (TyG) index, a simple and reliable surrogate marker of insulin resistance (IR), has garnered increasing attention in metabolic research. Although IR is mechanistically linked to carcinogenesis through multiple pathways, including chronic inflammation, hyperinsulinemia‐driven pro‐mitogenic signaling, and altered adipokine secretion, the specific utility of the TyG index for cancer risk assessment remains unclear. This systematic review examines whether the TyG index shows consistent associations across cancer types and holds value as an independent risk predictor beyond established metabolic syndrome components.

**Methods:**

We systematically searched PubMed, Embase, and Web of Science databases from 2008 (the year the TyG index was established as an IR marker) to December 31, 2024, for studies on the TyG index‐cancer association. Cohort, cross‐sectional, and case–control studies were included. Using meta‐analysis, we pooled effect sizes and conducted subgroup analyses by gender, region, population source, and study design. Trial sequential analysis (TSA) evaluated evidence reliability.

**Results:**

This meta‐analysis incorporated a total of 20 eligible studies. Our findings demonstrated that elevated TyG index levels were significantly associated with increased risks of various malignancies, including digestive system cancers (OR: 1.22, 95% CI 1.13–1.31), urogenital system cancers (OR: 2.04, 95% CI 1.53–2.71), and breast cancer (OR: 1.64, 95% CI 1.49–1.80) when compared to lower TyG index levels. These associations remained consistent across all pre‐specified subgroup analyses stratified by study characteristics. Furthermore, TSA confirmed sufficient statistical power for definitive conclusions.

**Conclusions:**

The consistent observed association between elevated TyG index and increased cancer risk highlights its potential as a candidate biomarker for further investigation. While these findings support the biological plausibility of insulin resistance in oncogenesis, current evidence—partially derived from observational studies—cannot establish causality or direct clinical utility. Future research should prioritize: (1) prospective validation of TyG index thresholds for cancer risk prediction, (2) mechanistic studies elucidating its role in tumor biology, and (3) assessment of its incremental value to existing risk stratification tools.

## 1 Introduction

The escalating global burden of cancer, reflected in rising incidence and mortality rates worldwide, represents a major public health challenge [1]. While cancer pathogenesis involves multifactorial etiology, growing evidence highlights the significant role of metabolic dysregulation in carcinogenesis [2]. In particular, insulin resistance—a hallmark of metabolic dysfunction—has emerged as a key pathophysiological link to increased risk of various malignancies [3].

TyG index, calculated as ln[fasting triglycerides (mg/dL) × fasting glucose (mg/dL)/2], serves as a reliable and easily obtainable surrogate marker for IR [4]. Substantial evidence has established its association with various pathological conditions, including cardiovascular [5], digestive, and genitourinary diseases [6]. The TyG index may similarly influence cancer risk through multiple biological pathways, such as promoting cell proliferation, inducing oxidative stress, and fostering a pro‐inflammatory microenvironment [3]. However, current research findings regarding the TyG index‐cancer association remain inconsistent, highlighting the need for a comprehensive systematic evaluation to consolidate the existing evidence. Additionally, we aim to elucidate the pathogenic role of IR‐related metabolic dysregulation in carcinogenesis while establishing a stronger theoretical foundation for cost‐effective cancer screening strategies. Furthermore, our findings may identify novel targets for therapeutic intervention in cancer prevention and management.

## 2 Methods

### 2.1 Search Strategy

We conducted a comprehensive systematic search of three major databases (PubMed, Embase, and Web of Science) from 2008 (the year the TyG index was established as an IR marker) through December 31, 2024. Our search strategy employed a combination of controlled vocabulary terms and free‐text words. Using PubMed as an illustrative example, the search algorithm incorporated the following key concepts: (“triglyceride glucose index” OR “TyG index” OR “triglyceride‐glucose index” OR “TyG”) AND (“cancer” OR “neoplasm” OR “malignancy” OR “tumor” OR “carcinoma”) AND (“odds ratio” OR “OR” OR “hazard ratio” OR “HR” OR “relative risk” OR “RR”) AND (“risk” OR “association” OR “relationship”). More detail in Appendix S1.

### 2.2 Literature Screening Process

The screening process was independently performed by two reviewers (Hongyu Li and Zijie Chen) under the supervision of a senior researcher (Xin Wang). The procedure comprised: (1) Title/abstract screening: Initial eligibility assessment by dual independent reviewers, with discrepancies resolved by the third arbitrator; (2) Full‐text review: Applying identical dual‐independent screening methodology; (3) Data extraction: Conducted using a standardized form after pilot testing on five publications (complete dataset in Appendix S2); (4) Risk of bias assessment: Independently executed by the two primary reviewers using predefined criteria (detailed evaluations in Appendix S3).

### 2.3 Inclusion and Exclusion Criteria

These studies included in the analysis met the following criteria: (1) The study adopted a cohort, cross‐sectional, or case–control design. (2) The participants enrolled in the trial are healthy at baseline. (3) The objective of the study was to evaluate the correlation between the TyG index and the risk of cancer. (4) The study provided HR, OR, RR, and their 95% confidence interval (CI) for different TyG index groups or every unit or standard deviation increase of the TyG index, or supplied relevant values that can calculate the 95% CI. (5) The effect size was adjusted using a multivariable analysis. (6) If multiple reports contain the same participants or overlapping individuals, priority should be given to the study with the largest sample size.

The exclusion criteria were defined as follows: (1) Conference abstracts presented at academic conferences. (2) Animal research, review papers, and Mendelian randomization studies. (3) Data related to expected results is either unavailable or not accessible.

### 2.4 Data Extraction and Quality Assessment

The We systematically extracted the following key variables from each included study: first author's name, publication year, and geographic region/country as basic study information; study design (cohort, case–control, or cross‐sectional), total sample size, and follow‐up duration (for longitudinal studies) as methodological characteristics; participant source (e.g., general population, hospital‐based), mean/median age and age range, and gender distribution as population demographics; TyG index categorization method (e.g., quartiles, tertiles, or continuous per 1‐unit/1‐SD increase) and reference category used for comparison as TyG index parameters; and effect estimates (HR, OR, or RR) from the most fully adjusted model with corresponding 95% CI and covariates included in the adjusted model as outcome measures.

We assessed study quality using standardized tools: the Newcastle‐Ottawa Scale (NOS) [7] for case–control and cohort studies (with scores ≥ 6 indicating high quality) and the Agency for Healthcare Research and Quality (AHRQ) [8] methodology for cross‐sectional studies (with scores ≥ 7 representing high quality).

### 2.5 Statistical Analysis

Of the twenty included studies, the majority reported odds ratios (ORs) as their primary effect measure, while a minority utilized hazard ratios (HRs). Consequently, we standardized all outcome measures to ORs with corresponding 95% CIs to quantify the TyG index‐cancer risk association. For categorical analyses, we compared the highest versus lowest TyG index categories, whereas for continuous analyses, we expressed effect sizes as per unit or per standard deviation increments in TyG index values.

We evaluated study heterogeneity using Cochran's *Q*‐test and quantified its magnitude through *I* ^2^ statistics. Based on the heterogeneity assessment results, we employed a random‐effects model when significant heterogeneity was detected (*Q*‐test *p* value < 0.05) [9]; otherwise, a fixed‐effects model was applied (*Q*‐test *p* value ≥ 0.05). To explore potential sources of heterogeneity, we conducted stratified analyses by gender, geographic region, study design, and participant source. Publication bias was systematically assessed using both Egger's and Begg's tests [10].

To address potential Type I errors and false‐positive results inherent in meta‐analyses—particularly given limited data availability and repeated testing in cumulative analyses—we conducted trial sequential analysis (TSA) [11]. The evidence was considered robust and conclusive if the cumulative *Z*‐curve crossed either the TSA monitoring boundary or the required information size (RIS) boundary [12]. If neither boundary was crossed, the observed associations were deemed inconclusive, indicating the need for additional studies to verify the findings.

Statistical analyses were carried out using the software Comprehensive Meta Analysis Version 3.0. A summarized forest plot was drawn using R version 4.3.3. TSA was conducted by Stata version 17.0.

## 3 Results

### 3.1 Literature Selection

**FIGURE 1:**
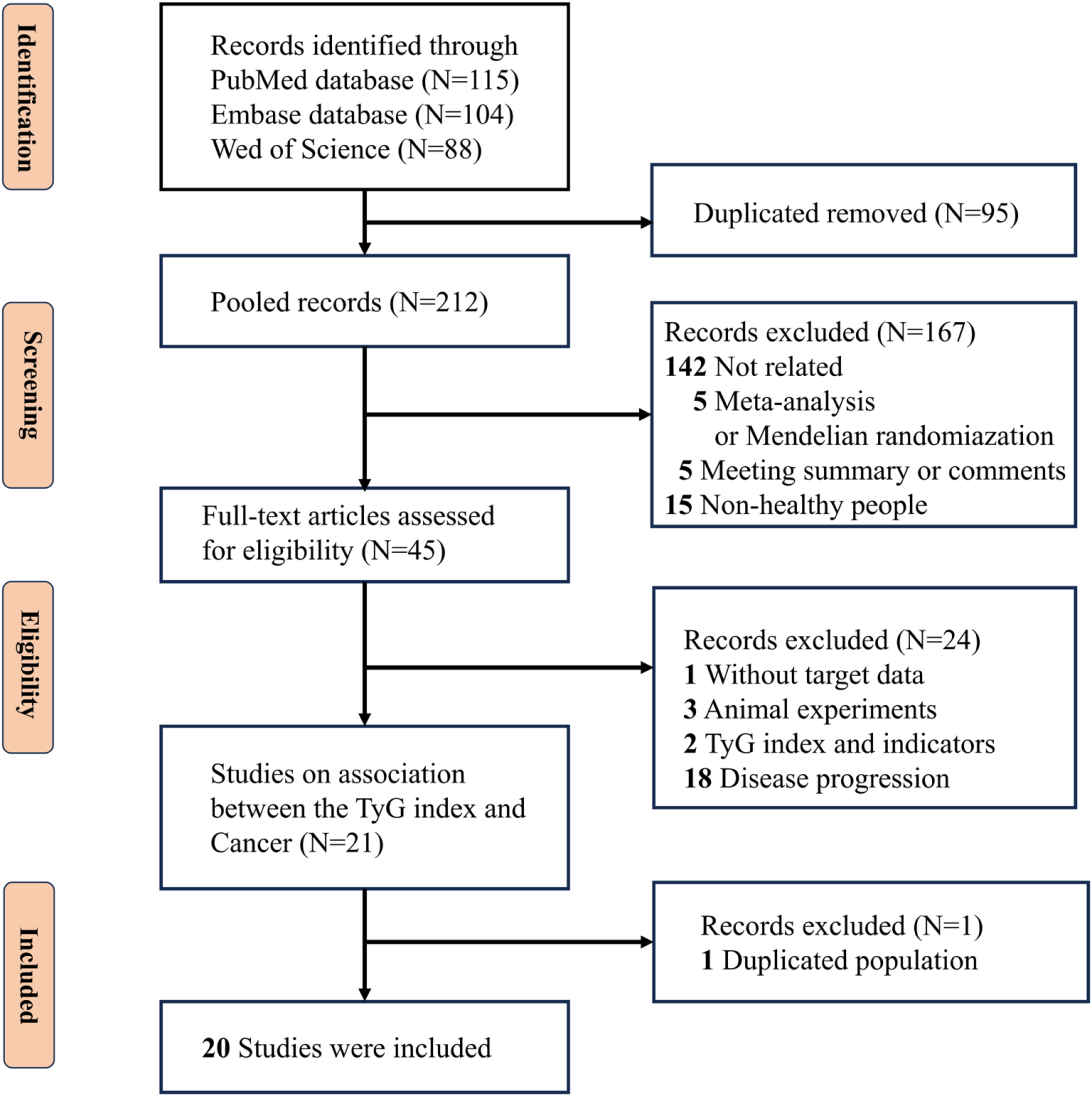
Flow chart of this study.

Our systematic search identified 307 potentially relevant articles across PubMed, Embase, and Web of Science databases. Following duplicate removal and rigorous screening based on predefined eligibility criteria, 20 studies were ultimately included for quantitative analysis of the TyG index‐cancer risk association. The complete study selection process, including reasons for exclusion at each stage, is detailed in Figure 1.

### 3.2 Study Characteristics

Our analysis incorporated 20 eligible studies examining TyG index‐cancer associations, which revealed the following distribution: Ten studies focused on digestive system malignancies (comprising seven colorectal cancer studies [13, 14, 15, 16, 17, 18, 19], along with one investigation each for liver cancer [14], pancreatic cancer [14], gastric cancer [20], and esophageal cancer [21]); two studies addressed respiratory system cancers [22, 23]; five examined urogenital cancers [14, 24, 25, 26, 27]; one studied endocrine system cancers [28]; and four investigated breast cancer [26, 29, 30, 31]. More details about the characteristics of the included studies are displayed in Appendices S2 and S3.

### 3.3 Main Meta‐Analysis

**FIGURE 2:**
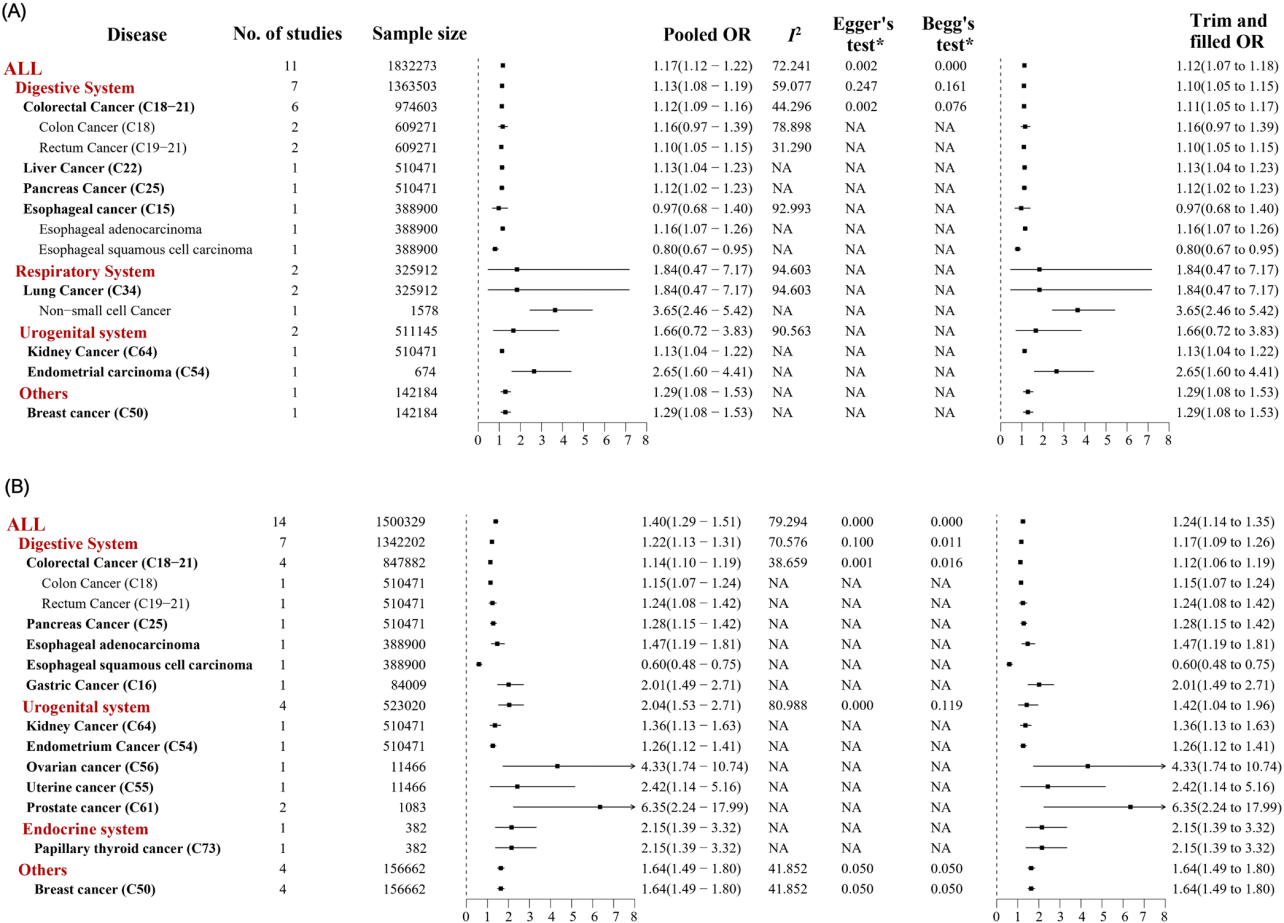
Forest plot of the association between the TyG index and cancer. (A) The association between the continuous triglyceride glucose (TyG) index and cancer risk reflects the change in cancer risk associated with each unit or standard deviation increase in the TyG index. (B) The association between the categorical TyG index and cancer risk reflects changes in cancer risk at higher levels of TyG compared to lower levels of TyG. *p value for publish bias.

When analyzing the TyG index as a categorical variable, our meta‐analysis demonstrated significant dose–response relationships between elevated TyG index levels and increased cancer risk. The pooled analysis revealed an overall 40% higher cancer risk among individuals in the highest TyG index category compared to the lowest (OR = 1.40, 95% CI: 1.29–1.51). Site‐specific analyses showed particularly strong associations for urogenital cancers (OR = 2.04, 95% CI: 1.53–2.71) and endocrine‐related cancers (OR = 2.15, 95% CI: 1.39–3.32), along with significant but more modest associations for digestive system cancers (OR = 1.22, 95% CI: 1.13–1.31) and breast cancer (OR = 1.64, 95% CI: 1.49–1.80) (Figure 2).

Analysis of TyG index as a continuous variable revealed significant positive associations for both digestive system cancers (pooled OR = 1.13, 95% CI: 1.08–1.19) and breast cancer (pooled OR = 1.29, 95% CI: 1.08–1.53). However, the limited number of studies examining respiratory system cancers (*n* = 2) and urogenital system cancers (*n* = 2) showed substantial heterogeneity (*I* ^2^ = 94.603% and 90.563%, respectively), resulting in non‐significant pooled estimates (*p* > 0.05 for both) (Figure 2).

Notably, a single study [21] reported an inverse association between TyG index and esophageal squamous cell carcinoma risk (OR = 0.8, 95% CI: 0.67–0.95). However, this contradicts the biological plausibility of insulin resistance increasing cancer risk through chronic inflammation and cellular proliferation. Moreover, seven other studies on upper gastrointestinal cancers (gastric/esophagogastric junction) consistently demonstrated positive associations, calling its reliability into question. This isolated inverse association likely reflects methodological limitations rather than true biological effects, requiring validation in larger prospective cohorts. Current evidence does not support a protective association between TyG index and esophageal squamous cell carcinoma.

### 3.4 Publication Bias

Both Egger's test and Begg's test indicated significant publication bias among studies examining the TyG index–colorectal cancer association. This bias persisted regardless of whether the TyG index was analyzed as a continuous (Egger's *p* = 0.002; Begg's *p* = 0.076) or categorical variable (Egger's *p* = 0.001; Begg's *p* = 0.016). However, sensitivity analysis using the trim‐and‐fill method revealed that the adjusted pooled odds ratios (continuous variable: OR = 1.11, 95% CI: 1.05–1.17; categorical variable: OR = 1.12, 95% CI: 1.06–1.19) did not differ substantially from the original estimates (continuous: OR = 1.12, 95% CI: 1.09–1.16; categorical: OR = 1.14, 95% CI: 1.10–1.19), suggesting that the observed publication bias did not significantly alter our conclusions (Figure 2).

### 3.5 Subgroup Analysis

Stratified analyses consistently demonstrated that elevated TyG index levels were significantly associated with increased cancer risk, regardless of variable type (continuous or categorical). When stratified by key covariates—including gender, geographic region, study design, and participant source—we observed moderate reductions in heterogeneity (*I* ^2^ decreased by 15%–40% across subgroups). Notably, this risk association remained statistically significant (all *p* < 0.05) in all stratified analyses (Table 1). When digestive as well as genitourinary cancers and breast cancer were analyzed separately, stratified according to the variables mentioned above, the conclusions remained the same (Appendix S4).

**TABLE 1:** Subgroup analysis for the associations between the TyG index and cancer.

Disease	No. of studies	Sample size	*I* ^2^ (%)	Pooled OR (95% CI)	*p* for interaction
(A) All cancer	11	1,832,273	72.241	1.171 (1.121–1.223)	
Study design					0.203
Case–control	2	3987	96.224	2.053 (0.685–6.158)	
Cohort	8	1,686,102	64.261	1.130 (1.080–1.182)	
Cross‐sectional	1	142,184	NA	1.290 (1.084–1.535)	
Geographic background					0.001
Asia	8	608,568	79.56	1.359 (1.200–1.540)	
Europe	3	1,223,705	53.325	1.089 (1.044–1.135)	
Source of participants					0.048
Employee	1	93,659	NA	1.190 (1.053–1.344)	
Health check‐up	5	273,925	90.66	1.672 (1.198–2.334)	
Multicenter	5	1,464,689	60.719	1.121 (1.072–1.172)	
Increase type					0.022
Not mentioned	4	594,272	76.893	1.339 (0.959–1.870)	
Per 1 SD	3	1,041,555	59.996	1.098 (1.051–1.147)	
Per 1 unit	4	196,446	82.953	1.376 (1.163–1.627)	
Sex					0.85
Female	3	850,614	0	1.195 (1.114–1.282)	
Male	3	850,614	58.082	1.181 (1.070–1.304)	
(B) All cancer	14	1,500,329	79.294	1.397 (1.292–1.51)	
Study design					0.00
Case–control	5	4298	82.716	2.361 (1.685–3.308)	
Cohort	5	1,318,382	70.824	1.205 (1.126–1.290)	
Cross‐sectional	4	177,649	19.783	1.741 (1.510–2.006)	
Geographic background					0.00
America	2	32,877	0	2.167 (1.767–2.657)	
Asia	9	567,699	83.104	1.547 (1.369–1.749)	
Europe	3	899,753	72.836	1.185 (1.076–1.305)	
Source of participants					0.269
Employee	1	93,659	NA	1.422 (1.205–1.704)	
Health check‐up	7	331,814	84.774	1.569 (1.333–1.846)	
Multicenter	6	1,074,856	75.353	1.345 (1.224–1.478)	
Sex					0.12
Female	3	532,306	63.898	2.016 (1.388–2.928)	
Male	1	93,659	NA	1.450 (1.209–1.740)	

*Note:* (A) Subgroup analysis of the association between the continuous TyG index and cancer. (B) Subgroup analysis of cancer risk with high levels of TyG index compared to low levels of the TyG index.

### 3.6 TSA for Evaluating the Reliability of the Evidence

**FIGURE 3:**
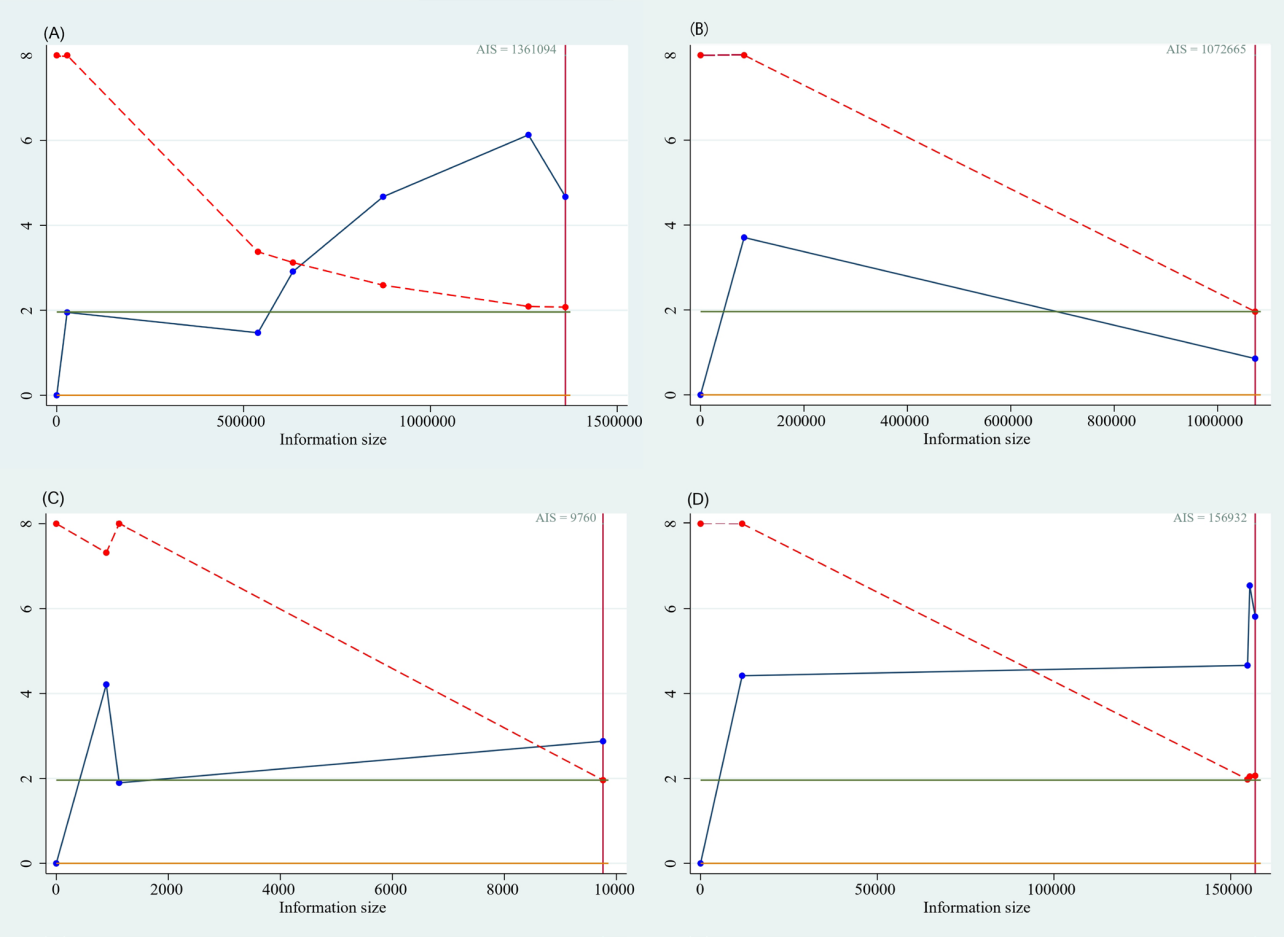
Trial sequential analysis (TSA) for evaluating the reliability of the association between the TyG index and cancer. (A) Continuous TyG index and the cancer of digestive system. (B) Categorical TyG index and the cancer of digestive system. (C) Categorical TyG index and the cancer of urogenital system. (D) Categorical TyG index and breast cancer. Monitoring boundaries calculated based on *observed effect size*. Does not preclude *residual confounding*.

For studies providing adequate data, we performed TSA to evaluate the robustness of observed associations. When analyzing TyG index categorically, the cumulative *Z*‐curves for digestive system, genitourinary, and breast cancers crossed both conventional (*Z* = 1.96, *p* = 0.05) and TSA monitoring boundaries (Figure 3). This indicates that the cumulative evidence reached sufficient information size to detect statistically significant associations with low false‐positive probability (< 5%).

## 4 Discussion

### 4.1 Summary

The TyG index, a well‐established marker of insulin resistance, emerges as a clinically relevant indicator for cancer risk stratification. Our comprehensive meta‐analysis of 20 observational studies demonstrates that elevated TyG index levels are significantly associated with an increased risk of various malignancies, including digestive system cancers (colorectal, pancreatic, and gastric cancers), genitourinary cancers (renal, endometrial, and ovarian cancers), and breast cancer. These associations remained robust across all subgroup analyses stratified by demographic and study characteristics. Compared to the prior meta‐analysis limited to six studies with a literature search up to July 2022 [32], our work provides three critical advancements. First, the inclusion of cohort, case–control, and cross‐sectional designs (versus exclusion of cross‐sectional data in earlier work) enhances generalizability while mitigating selection bias. Second, our updated synthesis through December 2024 incorporates recent large‐scale studies, capturing the accelerated evolution of evidence in this field. Third, the inclusion of multiple cancer types enabled stratified analyses to explore site‐specific associations—an essential advancement given the heterogeneous pathophysiology of malignancies. In contrast, the limited sample size of the previous meta‐analysis precluded robust subgroup investigations. Furthermore, while prior research did not employ TSA to rigorously evaluate the robustness of observed associations, our TSA suggests sufficient evidence for the association (false‐positive probability < 5%). However, methodological heterogeneity and potential biases warrant future prospective validation of its clinical predictive value.

### 4.2 Mechanisms

The elevated TyG index demonstrates a significant association with increased risk of digestive system malignancies, including colorectal, pancreatic, hepatic, and gastric cancers. This association may be mediated through multiple interrelated pathophysiological mechanisms: (1) Chronic inflammatory cascade: Insulin resistance induces adipose tissue dysfunction, triggering sustained release of pro‐inflammatory cytokines (TNF‐α, IL‐6) that establish a chronic low‐grade inflammatory state conducive to tumorigenesis [33, 34]. Through NF‐κB pathway activation, these inflammatory signals upregulate oncogenes (Cyclin D1, c‐Myc) while suppressing tumor suppressors (p53) [14, 35]. (2) Metabolic derangements: Hyperglycemia and elevated free fatty acids induce mitochondrial dysfunction, generating excessive reactive oxygen species that cause oxidative DNA damage and genomic instability. Concurrently, hypertriglyceridemia exacerbates lipotoxicity, particularly in hepatocytes, through NF‐κB‐mediated inflammatory responses [36]. (3) Microbiome dysregulation: Insulin resistance alters gut microbiota composition, characterized by increased pathogenic species (e.g., *Clostridium perfringens* ) [37] and decreased beneficial SCFA‐producing bacteria. This dysbiosis compromises intestinal barrier integrity and promotes localized inflammation [38]. (4) Direct mitogenic effects: Hyperinsulinemia stimulates excessive pancreatic β‐cell proliferation [39], potentially synergizing with oncogenic mutations (e.g., K‐RAS) [40, 41] in carcinogenesis. These mechanisms collectively create a pro‐tumorigenic microenvironment through interconnected metabolic, inflammatory, and genomic instability pathways.

Elevated TyG index promotes urogenital system cancers through similar metabolic‐inflammatory mechanisms as digestive cancers, with additional endocrine effects: (1) Hormonal dysregulation: Insulin resistance increases androgens (e.g., in PCOS) [42] and bioavailable estrogen (via sex hormone‐binding globulin [SHBG] suppression) and creates a pro‐tumor environment for endometrial/ovarian cancers [43]. (2) Metabolic effects: Visceral fat‐derived adipokines exacerbate hormonal imbalances. Chronic inflammation and oxidative stress damage genitourinary epithelia [44]. These endocrine‐metabolic interactions particularly drive hormone‐sensitive urogenital system malignancies.

The TyG index demonstrates a similar association with breast cancer risk, primarily mediated through estrogen pathway activation. Specifically, insulin resistance reduces SHBG production [45], resulting in elevated bioavailable estrogen levels that promote mammary epithelial proliferation and tumorigenesis [46].

### 4.3 Clinical Implications

Our findings demonstrate that the TyG index is a practical and cost‐effective biomarker for cancer risk assessment, requiring only routine blood glucose and triglyceride measurements. We recommend its integration into standard health screenings and cancer prediction models to identify high‐risk individuals for targeted prevention strategies.

### 4.4 Limitations

This study has several limitations: (1) The small number of studies on certain cancers (e.g., respiratory cancers) precludes definitive conclusions, warranting further investigation; (2) While some studies examined diagnostic utility, comprehensive evaluation of clinical applications requires additional evidence synthesis; (3) Notably, some subgroup analyses showed persistently high heterogeneity (*I* ^2^ > 90%). We consistently applied random‐effects models to address this. Given the limited sample sizes in high‐heterogeneity subgroups and underlying clinical heterogeneity (population characteristics/study designs), we restricted interpretation to qualitative consistency in association direction (i.e., the consistently positive relationship between elevated TyG index and cancer risk). These findings suggest the TyG index‐cancer risk association may involve multiple modulating factors, warranting validation through future prospective studies with standardized designs. (4) We fully acknowledge the current lack of consensus regarding TyG index thresholds in oncology. Our analysis intentionally preserved this real‐world heterogeneity in threshold definitions. Concurrently, we transparently documented the specific threshold methodologies employed in all included studies (detailed in Appendix S2). This approach underscores that establishing robust thresholds and identifying potential TyG inflection points represents both a current limitation and a significant opportunity for future clinical oncology practice. (5) As an observational study synthesis, causal inferences cannot be made, which also prevented TSA for all‐cancer outcomes.

## 5 Conclusion

In summary, this systematic review of observational studies demonstrates consistent positive associations between elevated TyG index and site‐specific cancer risks (digestive, genitourinary, breast), with magnitude varying across cancer types. While biologically plausible through alignment with insulin resistance pathways, these associations require prospective validation to establish causality, define cancer‐specific thresholds, and clarify their incremental value in cancer screening frameworks.

## Author Contributions

**Hongyu Li:** conceptualization (equal), data curation (lead), methodology (lead), software (lead), visualization (lead), writing – original draft (lead), writing – review and editing (lead). **Zijie Chen:** conceptualization (equal), data curation (lead), methodology (equal), project administration (equal), software (equal), visualization (equal), writing – original draft (lead), writing – review and editing (lead). **Guoheng Jiang:** conceptualization (supporting), methodology (supporting), software (supporting), writing – original draft (equal), writing – review and editing (supporting). **Wenqian Yu:** data curation (equal), methodology (supporting), writing – original draft (supporting), writing – review and editing (supporting). **Jing Luo:** conceptualization (supporting), writing – original draft (supporting). **Shiyi Li:** conceptualization (supporting), data curation (supporting), writing – original draft (supporting). **Linjun Xie:** methodology (equal), writing – original draft (supporting). **Xuan Bai:** data curation (supporting), methodology (supporting), writing – original draft (supporting). **Yiting Xu:** data curation (supporting), methodology (supporting), writing – original draft (supporting). **Yi Jiang:** conceptualization (supporting), writing – original draft (supporting). **Menglin He:** conceptualization (supporting), writing – original draft (supporting). **Min Mao:** conceptualization (supporting), methodology (lead), software (equal), validation (equal), visualization (equal), writing – original draft (equal). **Xin Wang:** conceptualization (lead), data curation (lead), formal analysis (lead), investigation (lead), methodology (lead), supervision (lead), validation (lead), writing – original draft (lead), writing – review and editing (lead).

## Ethics Statement

The authors have nothing to report.

## Conflicts of Interest

The authors declare no conflicts of interest.

## Supporting information


**Data S1:** PRISMA 2020 checklist.

**Appendix S1:** Literature search and study selection materials. A1: Complete search strategies (PubMed, Embase, Web of Science). A2: Study selection criteria. A3: Detailed data collection content.

**Appendix S2:** Characteristics of included studies. This appendix presents the detailed data extracted from each study included in the systematic review.

**Appendix S3:** Quality assessment of included studies. This appendix details the methodological quality for each included study, generated using the [Newcastle‐Ottawa Scale (NOS) and the Agency for Healthcare Research and Quality (AHRQ)] tool. It includes the rationale for each judgment and a summary of scores.

**Appendix S4:** Subgroup analyses of the association between the triglyceride‐glucose (TyG) index and cancer risk. (A) Subgroup analyses for the association between the TyG index as a continuous variable and cancer risk. (B) Subgroup analysis for the association between high versus low levels of the TyG index (categorical variable) and cancer risk.

## Data Availability

The original contributions presented in the study are included in the article/Supporting Information; further inquiries can be directed to the corresponding author.
