# Supplementary material for: The Associations Between the TyG Index and the Risk of Cancer—A Systematic Review and Meta‐Analysis
Source: Cancer Med. 2025 Oct 2;14(19):e71232. doi: 10.1002/cam4.71232 (PMC12489462; doi:10.1002/cam4.71232)
Supplement: Supplementary file 2 — Appendix S1: Literature search and study selection materials. A1: Complete search strategies (PubMed, Embase, Web of Science). A2: Study selection criteria. A3: Detailed data collection content. [file CAM4-14-e71232-s004.docx]

**Appendix File: The materials for the literature search, study selection and data extradition.**

**1. Literature search**

PubMed, Embase, and Web of Science databases were systematically searched for studies published up to December 31, 2024. The search formulas for each database are respectively:

**PubMed:**

("triglyceride glucose index" OR "TyG index" OR "triglyceride-glucose index" OR "TyG")

AND ("cancer" OR "neoplasm" OR "malignancy" OR "tumor" OR "carcinoma")

AND ("odds ratio" OR "OR" OR "hazard ratio" OR "HR" OR "relative risk" OR "RR")

AND ("risk" OR "association" OR "relationship")

Or search by subject line: "Insulin Resistance"[Mesh]) AND ("Neoplasms"[Mesh] OR "Cancer"[Mesh])

**Embase:**

('triglyceride glucose index'/exp OR 'TyG index'/exp OR 'triglyceride-glucose index'/exp OR 'TyG'/exp)

AND ('cancer'/exp OR 'neoplasm'/exp OR 'malignancy'/exp OR 'tumor'/exp OR 'carcinoma'/exp)

AND ('odds ratio'/exp OR 'OR'/exp OR 'hazard ratio'/exp OR 'HR'/exp OR 'relative risk'/exp OR 'RR'/exp)

AND ('risk'/exp OR 'association'/exp OR 'relationship'/exp)

**Web of Science:**

TS=("triglyceride glucose index" OR "TyG index" OR "triglyceride-glucose index" OR "TyG")

AND TS=("cancer" OR "neoplasm" OR "malignancy" OR "tumor" OR "carcinoma")

AND TS=("odds ratio" OR "OR" OR "hazard ratio" OR "HR" OR "relative risk" OR "RR")

AND TS=("risk" OR "association" OR "relationship")

**2. Study selection**

These studies included in the analysis met the following criteria: 1) The study adopted a cohort, cross-sectional or case-control design. 2) The participants enrolled in the trial are healthy at baseline. 3) The objective of the study was to evaluate the correlation between the TyG index and the risk of cancer. 4) The study provided HR, OR, RR and their 95% confidence interval (CI) for different TyG index groups or every unit or standard deviation increase of TyG index, or supplied relevant values that can calculate the 95% CI. 5) The effect size was adjusted using a multivariable analysis. 6) If multiple reports contain the same participants or overlapping individuals, priority should be given to the study with the largest sample size.

The exclusion criteria were defined as follows: 1) Conference abstracts presented at academic conferences. 2)Animal research, review papers, and Mendelian randomization study. 3) Data related to expected results is either unavailable or not accessible.

**3. Data abstraction**

The following data were extracted: 1) the first author; 2) year of publication; 3) geographic background; 4) study design; 5) sample size; 6) participants’ characteristics such as source of population, mean age, gender; 7) Categories of the TyG index (1 unit or 1 standard deviation); 8) hazard ratio (HR) or odds ratio (OR) or relative risk (RR)from the most adjusted model (with 95% CI); 8) follow-up period.
