# Supplementary material for: The Associations Between the TyG Index and the Risk of Cancer—A Systematic Review and Meta‐Analysis
Source: Cancer Med. 2025 Oct 2;14(19):e71232. doi: 10.1002/cam4.71232 (PMC12489462; doi:10.1002/cam4.71232)
Supplement: Supplementary file 3 — Appendix S2: Characteristics of included studies. This appendix presents the detailed data extracted from each study included in the systematic review. [file CAM4-14-e71232-s003.pdf]

Title	Study ID	PMID/DOI	Publish Year	Publish Country	Study Type	Period	Participants	Disease	Subgroup	Esposure group	Control group	Total	Case	Male	Female	Mean age	Follow-up (years)	Effect	Effect size	LCI(95%	UCI(95%	Covariate	NOS scores
Triglyceride–glucose index (TyG index) is a predictor of incident colorectal cancer: a population-based longitudinal study	Okamura, T 2020	32709256	2020	Japan	Retrospective Cohort Study	2004-2013	This was a retrospective sub-analysis of the ongoing cohort study named NAGALA (NAfld in the Gifu Area, Longitudinal Analysis) study,which is a medical checkup program and a cohort investigation that has been ongoing at Asahi University Hospital (Gifu, Japan).	Colorectal Cancer	NA	Increase in TyG	NA	27921	116	16434	11487	45.7±10.1	Median 4.4	HR	1.38	1	1.91	Adjusted for sex, age, body mass index, smoking status, alcohol consumption, exercise, systolic blood pressure and serum creatinine	5
The triglyceride-glucose index as a measure of insulin resistance and risk of obesity-related cancers	Fritz, J 2020-1	30945727	2020	Austria	Cohort Study	1972-2014	From the Metabolic Syndrome and Cancer Project (Me-Can) 2.0, a pooling of six population-based cohorts	Colon Cancer (ICD10-C18)	NA	Per 1 SD increase in TyG	NA	510471	4032	257968	252503	43.1±10.6	Median 17.2	HR	1.07	1.03	1.1	Adjusted for baseline age, sex, smoking status, fasting status, cohort and decade of birth.,additionally for BMI category.	9
The triglyceride-glucose index as a measure of insulin resistance and risk of obesity-related cancers	Fritz, J 2020-2	30945727	2020	Austria	Cohort Study	1972-2014	From the Metabolic Syndrome and Cancer Project (Me-Can) 2.0, a pooling of six population-based cohorts	Rectum Cancer (ICD10-C19-21)	NA	Per 1 SD increase in TyG	NA	510471	2430	257968	252503	43.1±10.6	Median 17.2	HR	1.09	1.04	1.14	Adjusted for baseline age, sex, smoking status, fasting status, cohort and decade of birth.,additionally for BMI category.	9
The triglyceride-glucose index as a measure of insulin resistance and risk of obesity-related cancers	Fritz, J 2020-3	30945727	2020	Austria	Cohort Study	1972-2014	From the Metabolic Syndrome and Cancer Project (Me-Can) 2.0, a pooling of six population-based cohorts	Liver Cancer (ICD10-C22)	NA	Per 1 SD increase in TyG	NA	510471	561	257968	252503	43.1±10.6	Median 17.2	HR	1.13	1.04	1.23	Adjusted for baseline age, sex, smoking status, fasting status, cohort and decade of birth.,additionally for BMI category.	9
The triglyceride-glucose index as a measure of insulin resistance and risk of obesity-related	Fritz, J 2020-4	30945727	2020	Austria	Cohort Study	1972-2014	From the Metabolic Syndrome and Cancer Project (Me-Can) 2.0, a pooling of six population-based	Pancreas (ICD10-C25)	NA	Per 1 SD increase in TyG	NA	510471	1368	257968	252503	43.1±10.6	Median 17.2	HR	1.12	1.06	1.19	Adjusted for baseline age, sex, smoking status, fasting status, cohort and decade of birth.,additionally for BMI	9
The triglyceride-glucose index as a measure of insulin resistance and risk of obesity-related	Fritz, J 2020-5	30945727	2020	Austria	Cohort Study	1972-2014	From the Metabolic Syndrome and Cancer Project (Me-Can) 2.0, a pooling of six population-based	Pancreas (ICD10-C25)	Male	Per 1 SD increase in TyG	NA	259768	776	257968	0	43.1±10.6	Median 17.2	HR	1.08	1	1.16	Adjusted for baseline age, sex, smoking status, fasting status, cohort and decade of birth.,additionally for BMI	9
The triglyceride-glucose index as a measure of insulin resistance and risk of obesity-related	Fritz, J 2020-6	30945727	2020	Austria	Cohort Study	1972-2014	From the Metabolic Syndrome and Cancer Project (Me-Can) 2.0, a pooling of six population-based	Pancreas (ICD10-C25)	Female	Per 1 SD increase in TyG	NA	252503	776	0	252503	43.1±10.6	Median 17.2	HR	1.19	1.09	1.31	Adjusted for baseline age, sex, smoking status, fasting status, cohort and decade of birth.,additionally for BMI	9
The triglyceride-glucose index as a measure of insulin resistance and risk of obesity-related	Fritz, J 2020-7	30945727	2020	Austria	Cohort Study	1972-2014	From the Metabolic Syndrome and Cancer Project (Me-Can) 2.0, a pooling of six population-based	Kidney Cancer (renal cell) (ICD10-C64)	NA	Per 1 SD increase in TyG	NA	510471	1347	257968	252503	43.1±10.6	Median 17.2	HR	1.13	1.07	1.2	Adjusted for baseline age, sex, smoking status, fasting status, cohort and decade of birth.,additionally for BMI	9
The triglyceride-glucose index as a measure of insulin resistance and risk of obesity-related	Fritz, J 2020-8	30945727	2020	Austria	Cohort Study	1972-2014	From the Metabolic Syndrome and Cancer Project (Me-Can) 2.0, a pooling of six population-based	Endometrium Cancer (ICD10-C54)	TyG Quartile 2 (8.1 to 8.4)	TyG Quartile 2 (8.1 to 8.4)	TyG Quartile 1 (<8.1)	60703	NA	0	60703	42.8±10.7	Median 17.2	HR	1.27	1.05	1.54	Adjusted for baseline age, sex, smoking status, fasting status, cohort and decade of birth.,additionally for BMI	9
The triglyceride-glucose index as a measure of insulin resistance and risk of obesity-related	Fritz, J 2020-9	30945727	2020	Austria	Cohort Study	1972-2014	From the Metabolic Syndrome and Cancer Project (Me-Can) 2.0, a pooling of six population-based	Endometrium Cancer (ICD10-C54)	TyG Quartile 4 (8.7 to 9.1)	TyG Quartile 4 (8.7 to 9.1)	TyG Quartile 1 (<8.1)	42143	NA	0	42143	44.4±10.2	Median 17.2	HR	1.28	1.06	1.54	Adjusted for baseline age, sex, smoking status, fasting status, cohort and decade of birth.,additionally for BMI	9
The triglyceride-glucose index as a measure of insulin resistance and risk of obesity-related cancers	Fritz, J 2020-10	30945727	2020	Austria	Cohort Study	1972-2014	From the Metabolic Syndrome and Cancer Project (Me-Can) 2.0, a pooling of six population-based cohorts	Endometrium Cancer (ICD10-C54)	TyG Quartile 5 (> 9.1)	TyG Quartile 5 (> 9.1)	TyG Quartile 1 (<8.1)	28078	NA	0	28078	44.9±9.4	Median 17.2	HR	1.22	1.01	1.41	Adjusted for baseline age, sex, smoking status, fasting status, cohort and decade of birth.,additionally for BMI category.	9
The triglyceride-glucose index as a measure of insulin resistance and risk of obesity-related cancers	Fritz, J 2020-11	30945727	2020	Austria	Cohort Study	1972-2014	From the Metabolic Syndrome and Cancer Project (Me-Can) 2.0, a pooling of six population-based cohorts	Pancreas (ICD10-C25)	TyG Quartile 3 (8.1 to 8.4)	TyG Quartile 3 (8.1 to 8.4)	TyG Quartile 1 (<8.1)	101581	NA	49640	52211	43.6±10.7	Median 17.2	HR	1.2	1	1.46	Adjusted for baseline age, sex, smoking status, fasting status, cohort and decade of birth.,additionally for BMI category.	9
The triglyceride-glucose index as a measure of insulin resistance and risk of obesity-related cancers	Fritz, J 2020-12	30945727	2020	Austria	Cohort Study	1972-2014	From the Metabolic Syndrome and Cancer Project (Me-Can) 2.0, a pooling of six population-based cohorts	Pancreas (ICD10-C25)	TyG Quartile 4 (8.7 to 9.1)	TyG Quartile 4 (8.7 to 9.1)	TyG Quartile 1 (<8.1)	101594	NA	59811	42143	44.4±10.2	Median 17.2	HR	1.27	1.05	1.53	Adjusted for baseline age, sex, smoking status, fasting status, cohort and decade of birth.,additionally for BMI category.	9
The triglyceride-glucose index as a measure of insulin resistance and risk of obesity-related cancers	Fritz, J 2020-13	30945727	2020	Austria	Cohort Study	1972-2014	From the Metabolic Syndrome and Cancer Project (Me-Can) 2.0, a pooling of six population-based cohorts	Pancreas (ICD10-C25)	TyG Quartile 5 (> 9.1)	TyG Quartile 5 (> 9.1)	TyG Quartile 1 (<8.1)	102125	NA	74047	28078	44.9±9.4	Median 17.2	HR	1.37	1.13	1.65	Adjusted for baseline age, sex, smoking status, fasting status, cohort and decade of birth.,additionally for BMI category.	9
The triglyceride-glucose index as a measure of insulin resistance and risk of obesity-related	Fritz, J 2020-14	30945727	2020	Austria	Cohort Study	1972-2014	From the Metabolic Syndrome and Cancer Project (Me-Can) 2.0, a pooling of six population-based	Colon Cancer (ICD10-C18)	TyG Quartile 4 (8.7 to 9.1)	TyG Quartile 4 (8.7 to 9.1)	TyG Quartile 1 (<8.1)	101594	NA	59811	42143	44.4±10.2	Median 17.2	HR	1.16	1.04	1.29	Adjusted for baseline age, sex, smoking status, fasting status, cohort and decade of birth.,additionally for BMI	9
The triglyceride-glucose index as a measure of insulin resistance and risk of obesity-related	Fritz, J 2020-15	30945727	2020	Austria	Cohort Study	1972-2014	From the Metabolic Syndrome and Cancer Project (Me-Can) 2.0, a pooling of six population-based	Colon Cancer (ICD10-C18)	TyG Quartile 5 (> 9.1)	TyG Quartile 5 (> 9.1)	TyG Quartile 1 (<8.1)	102125	NA	74047	28078	44.9±9.4	Median 17.2	HR	1.14	1.03	1.27	Adjusted for baseline age, sex, smoking status, fasting status, cohort and decade of birth.,additionally for BMI	9
The triglyceride-glucose index as a measure of insulin resistance and risk of obesity-related cancers	Fritz, J 2020-16	30945727	2020	Austria	Cohort Study	1972-2014	From the Metabolic Syndrome and Cancer Project (Me-Can) 2.0, a pooling of six population-based cohorts	Pancreas (ICD10-C25)	TyG Quartile 4;Female (8.7 to 9.1)	TyG Quartile 4;Female (8.7 to 9.1)	TyG Quartile 1 (<8.1)	42143	NA	0	42143	44.4±10.2	Median 17.2	HR	1.54	1.13	2.08	Adjusted for baseline age, sex, smoking status, fasting status, cohort and decade of birth.,additionally for BMI category.	9
The triglyceride-glucose index as a measure of insulin resistance and risk of obesity-related cancers	Fritz, J 2020-17	30945727	2020	Austria	Cohort Study	1972-2014	From the Metabolic Syndrome and Cancer Project (Me-Can) 2.0, a pooling of six population-based cohorts	Pancreas (ICD10-C25)	TyG Quartile 5;Female (> 9.1)	TyG Quartile 5;Female (> 9.1)	TyG Quartile 1 (<8.1)	28078	NA	0	28078	44.9±9.4	Median 17.2	HR	1.58	1.16	2.14	Adjusted for baseline age, sex, smoking status, fasting status, cohort and decade of birth.,additionally for BMI category.	9
The triglyceride-glucose index as a measure of insulin resistance and risk of obesity-related	Fritz, J 2020-18	30945727	2020	Austria	Cohort Study	1972-2014	From the Metabolic Syndrome and Cancer Project (Me-Can) 2.0, a pooling of six population-based	Rectum Cancer (ICD10-C19-21)	TyG Quartile 5 (> 9.1)	TyG Quartile 5 (> 9.1)	TyG Quartile 1 (<8.1)	102125	NA	74047	28078	44.9±9.4	Median 17.2	HR	1.24	1.08	1.42	Adjusted for baseline age, sex, smoking status, fasting status, cohort and decade of birth.,additionally for BMI	9
The triglyceride-glucose index as a measure of insulin resistance and risk of obesity-related	Fritz, J 2020-19	30945727	2020	Austria	Cohort Study	1972-2014	From the Metabolic Syndrome and Cancer Project (Me-Can) 2.0, a pooling of six population-based	Kidney Cancer (renal cell) (ICD10-C64)	TyG Quartile 5 (> 9.1)	TyG Quartile 5 (> 9.1)	TyG Quartile 1 (<8.1)	102125	NA	74047	28078	44.9±9.4	Median 17.2	HR	1.36	1.13	1.63	Adjusted for baseline age, sex, smoking status, fasting status, cohort and decade of birth.,additionally for BMI	9
Association between triglyceride-glucose index and gastric carcinogenesis: a health	Kim, Y. M. 2022-1	34355281	2022	Korea	Retrospective Cohort Study	2006-2020	The electronic medical records of 131,239 Korean patients who underwent upper endoscopy at the medical center, Gangnam Severance	Gastric Cancer	TyG Quartile 2 (8.80 to 9.18)	TyG Quartile 2 (8.80 to 9.18)	TyG Quartile 1 (control n=21006,case n=24) 6.45 to 8.80	21287	38	NA	NA	48.6±11.4	14	OR	1.619	1.101	2.812	Adjusted for baseline age, sex, smoking status, fasting glucose, BMI, height,weight,SBP,DBP,Obesity,al	7
Association between triglyceride-glucose index and gastric carcinogenesis: a health checkup cohort study	Kim, Y. M. 2022-2	34355281	2022	Korea	Retrospective Cohort Study	2006-2020	The electronic medical records of 131,239 Korean patients who underwent upper endoscopy at the medical center, Gangnam Severance Hospital,	Gastric Cancer	TyG Quartile 3 (9.18 to 9.61)	TyG Quartile 3 (9.18 to 9.61)	TyG Quartile 1 (control n=21006,case n=24) 6.45 to 8.80	21058	54	NA	NA	48.6±11.4	14	OR	2.18	1.288	3.691	Adjusted for baseline age, sex, smoking status, fasting glucose, BMI, height,weight,SBP,DBP,Obesity,al cohol	7
Association between triglyceride-glucose index and gastric carcinogenesis: a health checkup cohort study	Kim, Y. M. 2022-3	34355281	2022	Korea	Retrospective Cohort Study	2006-2020	The electronic medical records of 131,239 Korean patients who underwent upper endoscopy at the medical center, Gangnam Severance Hospital, in Seoul, Korea	Gastric Cancer	TyG Quartile 4 (9.61 to 13.03)	TyG Quartile 4 (9.61 to 13.03)	TyG Quartile 1 (control n=21006,case n=24) 6.45 to 8.80	20664	70	NA	NA	48.6±11.4	14	OR	2.363	1.391	4.014	Adjusted for baseline age, sex, smoking status, fasting glucose, BMI, height,weight,SBP,DBP,Obesity,al cohol consumption,DM,hepertension,	7
Association between the TyG index and TG/ HDL-C ratio as insulin resistance markers and the risk of colorectal cancer	Liu, T 2022-1	36138391	2022	China	Prospective cohort study	2006-2019	This current study came from the Kailuan study (Chinese Clinical Trial Registry number: ChiCTRTNRC-11001489), which is a prospective cohort study of current and retired Kailuan Group employees in Tangshan City, Hebei	Colorectal Cancer （ICD10-C18-21)	NA	Per 1 unit increase in TyG	NA	93659	593	74671	18988	51.44± 12.45	Median 13.02	HR	1.19	1.05	1.34	Adjusted for age (every 10years), sex, family income, educational background, marital status, WC, TC, smoking status, drinking status, physical activity, sedentary lifestyle, tea consumption, salt intake, high-fat diet, hypertension,	7
Association between the TyG index and TG/ HDL-C ratio as insulin resistance markers and the risk of colorectal cancer	Liu, T 2022-2	36138391	2022	China	Prospective cohort study	2006-2019	This current study came from the Kailuan study (Chinese Clinical Trial Registry number: ChiCTRTNRC-11001489), which is a prospective cohort study of current and retired Kailuan Group employees in Tangshan City, Hebei	Colorectal Cancer （ICD10-C18-21)	TyG Quartile 3 (8.58 to 9.06)	TyG Quartile 3 (8.58 to 9.06)	TyG Quartile 1 ,case n=111) <=8.19	NA	170	NA	NA	51.44± 12.45	Median 13.02	HR	1.36	1.06	1.76	Adjusted for age (every 10years), sex, family income, educational background, marital status, WC, TC, smoking status, drinking status, physical activity, sedentary lifestyle, tea consumption, salt intake, high-fat diet, hypertension,	7
Association between the TyG index and TG/ HDL-C ratio as insulin resistance markers and the risk of colorectal cancer	Liu, T 2022-3	36138391	2022	China	Prospective cohort study	2006-2019	This current study came from the Kailuan study (Chinese Clinical Trial Registry number: ChiCTRTNRC-11001489), which is a prospective cohort study of current and retired Kailuan Group employees in Tangshan City, Hebei	Colorectal Cancer （ICD10-C18-21)	TyG Quartile 4 (>=9.06)	TyG Quartile 4 (>=9.06)	TyG Quartile 1 ,case n=111) <=8.19	NA	181	NA	NA	51.44± 12.45	Median 13.02	HR	1.5	1.19	1.91	Adjusted for age (every 10years), sex, family income, educational background, marital status, WC, TC, smoking status, drinking status, physical activity, sedentary lifestyle, tea consumption, salt intake, high-fat diet, hypertension,	7
Association between the TyG index and TG/ HDL-C ratio as insulin resistance markers and the risk of colorectal cancer	Liu, T 2022-4	36138391	2022	China	Prospective cohort study	2006-2019	This current study came from the Kailuan study (Chinese Clinical Trial Registry number: ChiCTRTNRC-11001489), which is a prospective cohort study of current and retired Kailuan Group employees in Tangshan City, Hebei	Colorectal Cancer （ICD10-C18-21)	TyG Quartile 3 (8.58 to 9.06); Male	TyG Quartile 3 (8.58 to 9.06)	TyG Quartile 1 ,case n=96) <=8.19	NA	152	NA	0	51.44± 12.45	Median 13.02	HR	1.43	1.1	1.85	Adjusted for age (every 10years), sex, family income, educational background, marital status, WC, TC, smoking status, drinking status, physical activity, sedentary lifestyle, tea consumption, salt intake, high-fat diet, hypertension,	7
Association between the TyG index and TG/ HDL-C ratio as insulin resistance markers and the risk of colorectal cancer	Liu, T 2022-5	36138391	2022	China	Prospective cohort study	2006-2019	This current study came from the Kailuan study (Chinese Clinical Trial Registry number: ChiCTRTNRC-11001489), which is a prospective cohort study of current and retired Kailuan Group employees in Tangshan City, Hebei	Colorectal Cancer （ICD10-C18-21)	TyG Quartile 4 (>=9.06); Male	TyG Quartile 4 (>=9.06)	TyG Quartile 1 ,case n=96) <=8.19	NA	154	NA	0	51.44± 12.45	Median 13.02	HR	1.47	1.14	1.9	Adjusted for age (every 10years), sex, family income, educational background, marital status, WC, TC, smoking status, drinking status, physical activity, sedentary lifestyle, tea consumption, salt intake, high-fat diet, hypertension,	7
Association between the TyG index and TG/ HDL-C ratio as insulin resistance markers and the risk of colorectal cancer	Liu, T 2022-6	36138391	2022	China	Prospective cohort study	2006-2019	This current study came from the Kailuan study (Chinese Clinical Trial Registry number: ChiCTRTNRC-11001489), which is a prospective cohort study of current and retired Kailuan Group employees in Tangshan City, Hebei	Colorectal Cancer （ICD10-C18-21)	TyG Quartile 3 (8.58 to 9.06); Age:18 to 45	TyG Quartile 3 (8.58 to 9.06)	TyG Quartile 1 ,case n=10) <=8.19	NA	22	NA	NA	18 to 45	Median 13.02	HR	2.28	1.02	5.08	Adjusted for age (every 10years), sex, family income, educational background, marital status, WC, TC, smoking status, drinking status, physical activity, sedentary lifestyle, tea consumption, salt intake, high-fat diet, hypertension,	7
Association between the TyG index and TG/ HDL-C ratio as insulin resistance markers and the risk of colorectal cancer	Liu, T 2022-7	36138391	2022	China	Prospective cohort study	2006-2019	This current study came from the Kailuan study (Chinese Clinical Trial Registry number: ChiCTRTNRC-11001489), which is a prospective cohort study of current and retired Kailuan Group employees in Tangshan City, Hebei	Colorectal Cancer （ICD10-C18-21)	TyG Quartile 4 (>=9.06); Age:18 to 45	TyG Quartile 4 (>=9.06)	TyG Quartile 1 ,case n=10) <=8.19	NA	22	NA	NA	18 to 45	Median 13.02	HR	2.33	1.07	5.07	Adjusted for age (every 10years), sex, family income, educational background, marital status, WC, TC, smoking status, drinking status, physical activity, sedentary lifestyle, tea consumption, salt intake, high-fat diet, hypertension,	7

Association between the TyG index and TG/ HDL-C ratio as insulin resistance markers and the risk of colorectal cancer	Liu, T 2022-8	36138391	2022	China	Prospective cohort study	2006-2019	This current study came from the Kailuan study (Chinese Clinical Trial Registry number: ChiCTRTNRC-11001489), which is a prospective cohort study of current and retired Kailuan Group employees in Tangshan City, Hebei	Colorectal Cancer （ICD10-C18-21)	TyG Quartile 4 (>=9.06); Age:45 to 65	TyG Quartile 4 (>=9.06)	TyG Quartile 1 ,case n=72) <=8.19	NA	110	NA	NA	45 to 65	Median 13.02	HR	1.35	1.01	1.81	Adjusted for age (every 10years), sex, family income, educational background, marital status, WC, TC, smoking status, drinking status, physical activity, sedentary lifestyle, tea consumption, salt intake, high-fat diet, hypertension,	7
Association between the TyG index and TG/ HDL-C ratio as insulin resistance markers and the risk of colorectal cancer	Liu, T 2022-9	36138391	2022	China	Prospective cohort study	2006-2019	This current study came from the Kailuan study (Chinese Clinical Trial Registry number: ChiCTRTNRC-11001489), which is a prospective cohort study of current and retired Kailuan Group employees in Tangshan City, Hebei	Colorectal Cancer （ICD10-C18-21)	TyG Quartile 4 (>=9.06); Age:>65,<98	TyG Quartile 4 (>=9.06)	TyG Quartile 1 ,case n=29) <=8.19	NA	49	NA	NA	65 to 98	Median 13.02	HR	1.62	1.02	2.59	Adjusted for age (every 10years), sex, family income, educational background, marital status, WC, TC, smoking status, drinking status, physical activity, sedentary lifestyle, tea consumption, salt intake, high-fat diet, hypertension,	7
Association between Surrogate Markers of Insulin Resistance and the Incidence of Colorectal Cancer in Korea: A Nationwide	Lee, J. Y 2024-1	38541854	2024	Korea	Retrospective Cohort Study	2009-2019	This retrospective population-based cohort study used claims data obtained from the National Health Insurance Service—National Health Screening Cohort (NHIS-HealS)	Colorectal Cancer	TyG Quartile 2 (8.5 ± 0.1)	TyG Quartile 2 (8.5 ± 0.1)	TyG Quartile 1 (8.0 ± 0.2) total=78613 case=1266	77345	1468	39296	38049	59.0±8.8	10	HR	1.08	1	1.16	Adjusted for age, sex, income level, residence, hypertension, diabetes, dyslipidemia, Charlson comorbidity index, body mass index, hemoglobin level, glomerular filtration rate,	8
Association between Surrogate Markers of Insulin Resistance and the Incidence of Colorectal Cancer in Korea: A Nationwide	Lee, J. Y 2024-2	38541854	2024	Korea	Retrospective Cohort Study	2009-2019	This retrospective population-based cohort study used claims data obtained from the National Health Insurance Service—National Health Screening Cohort (NHIS-HealS)	Colorectal Cancer	TyG Quartile3 (8.8 ± 0.1)	TyG Quartile 3 (8.5 ± 0.1)	TyG Quartile 1 (8.0 ± 0.2) total=78613 case=1266	78955	1606	43783	35172	59.4±8.8	10	HR	1.1	1.02	1.19	Adjusted for age, sex, income level, residence, hypertension, diabetes, dyslipidemia, Charlson comorbidity index, body mass index, hemoglobin level, glomerular filtration rate,	8
Association between Surrogate Markers of Insulin Resistance and the Incidence of Colorectal Cancer in Korea: A Nationwide	Lee, J. Y 2024-3	38541854	2024	Korea	Retrospective Cohort Study	2009-2019	This retrospective population-based cohort study used claims data obtained from the National Health Insurance Service—National Health Screening Cohort (NHIS-HealS)	Colorectal Cancer	TyG Quartile 4 (9.4 ± 0.3)	TyG Quartile 4 (8.5 ± 0.1)	TyG Quartile 1 (8.0 ± 0.2) total=78613 case=1266	79228	1772	50396	28832	59.1±8.7	10	HR	1.16	1.07	1.25	Adjusted for age, sex, income level, residence, hypertension, diabetes, dyslipidemia, Charlson comorbidity index, body mass index, hemoglobin level, glomerular filtration rate,	8
Association between Surrogate Markers of Insulin Resistance and the Incidence of Colorectal Cancer in Korea: A Nationwide	Lee, J. Y 2024-4	38541854	2024	Korea	Retrospective Cohort Study	2009-2019	This retrospective population-based cohort study used claims data obtained from the National Health Insurance Service—National Health Screening Cohort (NHIS-HealS)	Colorectal Cancer	Male	Increase in TyG	NA	169332	NA	169332	0	NA	10	HR	1.16	1.05	1.27	Adjusted for age, sex, income level, residence, hypertension, diabetes, dyslipidemia, Charlson comorbidity index, body mass index, hemoglobin level, glomerular filtration rate,	8
Association between Surrogate Markers of Insulin Resistance and the Incidence of Colorectal Cancer in Korea: A Nationwide	Lee, J. Y 2024-5	38541854	2024	Korea	Retrospective Cohort Study	2009-2019	This retrospective population-based cohort study used claims data obtained from the National Health Insurance Service—National Health Screening Cohort (NHIS-HealS)	Colorectal Cancer	Female	Increase in TyG	NA	144809	NA	0	144809	NA	10	HR	1.19	1.04	1.35	Adjusted for age, sex, income level, residence, hypertension, diabetes, dyslipidemia, Charlson comorbidity index, body mass index, hemoglobin level, glomerular filtration rate,	8
Association between Surrogate Markers of Insulin Resistance and the Incidence of Colorectal Cancer in Korea: A Nationwide	Lee, J. Y 2024-6	38541854	2024	Korea	Retrospective Cohort Study	2009-2019	This retrospective population-based cohort study used claims data obtained from the National Health Insurance Service—National Health Screening Cohort (NHIS-HealS)	Colorectal Cancer	Age :40 TO 65	Increase in TyG	NA	241343	NA	NA	NA	40-65	10	HR	1.11	1,01	1.23	Adjusted for age, sex, income level, residence, hypertension, diabetes, dyslipidemia, Charlson comorbidity index, body mass index, hemoglobin level, glomerular filtration rate,	8
Triglyceride-glucose index (TyG index) and endometrial carcinoma risk: A retrospective cohort study	Shi, H 2024	37555382	2024	China	Retrospective Cohort Study	2019-20221	Included patients who were clinically and pathologically diagnosed with EC or EAH at the Affiliated Drum Tower Hospital of Nanjing University Medical School	Endometrial carcinoma	NA	Increase in TyG	NA	674	300	0	674	54.3 ± 13.[illegible]	NA	OR	2.65	1.6	4.41	Adjusted for age, abortion, age at first menarche, BMI, CRP, hypertension, HDL-C, LDL-C, menopausal status, neutrophil count,	5
Association between four insulin resistance surrogates and the risk of esophageal cancer: a prospective cohort study	Yang, C 2024-1	39180548	2024	Germany	Prospective cohort study	2006-	UK biobank	Esophageal cancer	NA	Per 1 SD increase in TyG	NA	388900	779	183526	205374	57.0 (50.0–63.0)	Average 13	HR	1.07	1	1.15	Adjuested for age, sex, ethnicity, Townsend deprivation index, Metabolic Equivalent of Task (MET), smoking status, alcohol status, diabetes mellitus (DM), hypertension, insulin, fasting time	7
Association between four insulin resistance surrogates and the risk of esophageal cancer: a prospective cohort study	Yang, C 2024-2	39180548	2024	Germany	Prospective cohort study	2006-	UK biobank	EAC: esophageal adenocarcinoma	NA	Per 1 SD increase in TyG	NA	388900	NA	183526	205374	57.0 (50.0–63.0)	Average 13	HR	1.16	1.07	1.26	Adjuested for age, sex, ethnicity, Townsend deprivation index, Metabolic Equivalent of Task (MET), smoking status, alcohol status, diabetes mellitus (DM), hypertension, insulin, fasting time	7
Association between four insulin resistance surrogates and the risk of esophageal cancer: a prospective cohort study	Yang, C 2024-3	39180548	2024	Germany	Prospective cohort study	2006-	UK biobank	ESCC: esophageal squamous cell carcinoma	NA	Per 1 SD increase in TyG	NA	388900	NA	183526	205374	57.0 (50.0–63.0)	Average 13	HR	0.8	0.67	0.95	Adjuested for age, sex, ethnicity, Townsend deprivation index, Metabolic Equivalent of Task (MET), smoking status, alcohol status, diabetes mellitus (DM), hypertension, insulin, fasting time	7
Association between four insulin resistance surrogates and the risk of esophageal cancer: a prospective cohort study	Yang, C 2024-4	39180548	2024	Germany	Prospective cohort study	2006-	UK biobank	ESCC: esophageal squamous cell carcinoma	TyG quartile 2	TyG quartile 2	TyG quartile 1	NA	NA	NA	NA	37-73	Average 13	HR	0.67	0.46	0.97	Adjuested for age, sex, ethnicity, Townsend deprivation index, Metabolic Equivalent of Task (MET), smoking status, alcohol status, diabetes mellitus (DM), hypertension, insulin, fasting time	7
Association between four insulin resistance surrogates and the risk of esophageal cancer: a prospective cohort study	Yang, C 2024-5	39180548	2024	Germany	Prospective cohort study	2006-	UK biobank	ESCC: esophageal squamous cell carcinoma	TyG quartile 3	TyG quartile 3	TyG quartile 1	NA	NA	NA	NA	37-73	Average 13	HR	0.57	0.39	0.85	Adjuested for age, sex, ethnicity, Townsend deprivation index, Metabolic Equivalent of Task (MET), smoking status, alcohol status, diabetes mellitus (DM), hypertension, insulin, fasting time	7
Association between four insulin resistance surrogates and the risk of esophageal cancer: a prospective cohort study	Yang, C 2024-6	39180548	2024	Germany	Prospective cohort study	2006-	UK biobank	ESCC: esophageal squamous cell carcinoma	TyG quartile 4	TyG quartile 4	TyG quartile 1	NA	NA	NA	NA	37-73	Average 13	HR	0.55	0.37	0.82	Adjuested for age, sex, ethnicity, Townsend deprivation index, Metabolic Equivalent of Task (MET), smoking status, alcohol status, diabetes mellitus (DM), hypertension, insulin, fasting time	7
Association between four insulin resistance surrogates and the risk of esophageal cancer: a prospective cohort study	Yang, C 2024-7	39180548	2024	Germany	Prospective cohort study	2006-	UK biobank	EAC: esophageal adenocarcinoma	TyG quartile 3	TyG quartile 3	TyG quartile 1	NA	NA	NA	NA	37-73	Average 13	HR	1.4	1.05	1.89	Adjuested for age, sex, ethnicity, Townsend deprivation index, Metabolic Equivalent of Task (MET), smoking status, alcohol status, diabetes mellitus (DM), hypertension, insulin, fasting time	7
Association between four insulin resistance surrogates and the risk of esophageal cancer: a prospective cohort study	Yang, C 2024-8	39180548	2024	Germany	Prospective cohort study	2006-	UK biobank	EAC: esophageal adenocarcinoma	TyG quartile 4	TyG quartile 4	TyG quartile 1	NA	NA	NA	NA	37-73	Average 13	HR	1.54	1.15	2.06	Adjuested for age, sex, ethnicity, Townsend deprivation index, Metabolic Equivalent of Task (MET), smoking status, alcohol status, diabetes mellitus (DM), hypertension, insulin, fasting time	7
Triglyceride-Glucose Index, Modifiable Lifestyle, and Risk of Colorectal Cancer: A Prospective Analysis of the Korean Genome and Epidemiology Study	Kityo, A 2024-1	39103728	2024	Korea	Prospective cohort study	2004-2020	From the Korean Genome and Epidemiology Study-Health Examinees cohort (KoGES-HEXA) of 173,202 participants who were recruited between 2004 and 2013 at 38 health examination centers and training hospitals located in eight	Colorectal Cancer(ICD10-C18-C21)	NA	Per 1 unit Increase in TyG	NA	98800	699	33045	65755	53.2±8.3	10.6±2	HR	1.28	1.12	1.46	Adjusted for r educational level, monthly income, smoking, drinking, regular physical exercise, age,sex,BMI, fruit and vegetable intake, and total red meat intake	8
Triglyceride-Glucose Index, Modifiable Lifestyle, and Risk of Colorectal Cancer: A Prospective Analysis of the Korean Genome and Epidemiology Study	Kityo, A 2024-2	39103728	2024	Korea	Prospective cohort study	2004-2020	From the Korean Genome and Epidemiology Study-Health Examinees cohort (KoGES-HEXA) of 173,202 participants who were recruited between 2004 and 2013 at 38 health examination centers and training hospitals located in eight	Colon Cancer (ICD10-C18)	NA	Per 1 unit Increase in TyG	NA	98800	422	33045	65755	53.2±8.3	10.6±2	HR	1.29	1.1	1.54	Adjusted for r educational level, monthly income, smoking, drinking, regular physical exercise, age,sex,BMI, fruit and vegetable intake, and total red meat intake	8
Triglyceride-Glucose Index, Modifiable Lifestyle, and Risk of Colorectal Cancer: A Prospective Analysis of the Korean Genome and Epidemiology Study	Kityo, A 2024-3	39103728	2024	Korea	Prospective cohort study	2004-2020	From the Korean Genome and Epidemiology Study-Health Examinees cohort (KoGES-HEXA) of 173,202 participants who were recruited between 2004 and 2013 at 38 health examination centers and training hospitals located in eight	Rectum Cancer (ICD10-C19-21)	NA	Per 1 unit Increase in TyG	NA	98800	277	33045	65755	53.2±8.3	10.6±2	HR	1.24	1.01	1.52	Adjusted for r educational level, monthly income, smoking, drinking, regular physical exercise, age,sex,BMI, fruit and vegetable intake, and total red meat intake	8
Triglyceride-Glucose Index, Modifiable Lifestyle, and Risk of Colorectal Cancer: A Prospective Analysis of the Korean Genome and Epidemiology Study	Kityo, A 2024-4	39103728	2024	Korea	Prospective cohort study	2004-2020	From the Korean Genome and Epidemiology Study-Health Examinees cohort (KoGES-HEXA) of 173,202 participants who were recruited between 2004 and 2013 at 38 health examination centers and training hospitals located in eight	Colorectal Cancer(ICD10-C18-C21)	Male	Per 1 unit Increase in TyG	NA	33045	340	33045	0	40-69	10.6±2	HR	1.34	1.12	1.6	Adjusted for r educational level, monthly income, smoking, drinking, regular physical exercise, age,sex,BMI, fruit and vegetable intake, and total red meat intake	8
Triglyceride-Glucose Index, Modifiable Lifestyle, and Risk of Colorectal Cancer: A Prospective Analysis of the Korean Genome and Epidemiology Study	Kityo, A 2024-5	39103728	2024	Korea	Prospective cohort study	2004-2020	From the Korean Genome and Epidemiology Study-Health Examinees cohort (KoGES-HEXA) of 173,202 participants who were recruited between 2004 and 2013 at 38 health examination centers and training hospitals located in eight	Colon Cancer (ICD10-C18)	Male	Per 1 unit Increase in TyG	NA	33045	189	33045	0	40-69	10.6±2	HR	1.36	1.05	1.4	Adjusted for r educational level, monthly income, smoking, drinking, regular physical exercise, age,sex,BMI, fruit and vegetable intake, and total red meat intake	8
Triglyceride-Glucose Index, Modifiable Lifestyle, and Risk of Colorectal Cancer: A Prospective Analysis of the Korean Genome and Epidemiology Study	Kityo, A 2024-6	39103728	2024	Korea	Prospective cohort study	2004-2020	From the Korean Genome and Epidemiology Study-Health Examinees cohort (KoGES-HEXA) of 173,202 participants who were recruited between 2004 and 2013 at 38 health examination centers and training hospitals located in eight	Colorectal Cancer(ICD10-C18-C21)	Female	Per 1 unit Increase in TyG	NA	65755	359	0	65755	40-69	10.6±2	HR	1.23	1.01	1.49	Adjusted for r educational level, monthly income, smoking, drinking, regular physical exercise, age,sex,BMI, fruit and vegetable intake, and total red meat intake	8
Triglyceride-Glucose Index Is Not Associated With Lung Cancer Risk: A Prospective Cohort Study in the UK Biobank	Wan, L.J 2021	34869022	2021	China	Prospective Cohort Study	2006-2018	UK Biobank	Lung cancer(ICD10 -C34)	NA	Increase in TyG	NA	324334	1593	143277	181057	38 to 73	Median 9.07	HR	0.911	0.64	1.182	Adjusted for age, sex, region, Townsend deprivation score, smoking status, alcohol intake frequency, body mass index, waist hip rate, and hypertension, plus fasting time, total cholesterol, low-density lipoprotein cholesterol, high-density	9
Association of triglyceride-glucose index with the risk of prostate cancer: a retrospective study	Li, T.Q 2023-1	37953784	2023	China	Case-control study	2020-2022	In this study, 300 patients with prostate cancer diagnosed pathologically by prostate puncture biopsy in the First Affiliated Hospital of Xinjiang Medical University between 2020 and 2022 were retrospectively collected, and 3,000 healthy people without chronic or	Prostate cancer(ICD10-C61)	TyG Quartile 2 (8.001 to 8.373)	TyG Quartile 2 (8.001 to 8.373)	TyG Quartile 1 (<8.0)	194	18	NA	NA	71.00 (64.25,78.75)	NA	OR	3.124	1.006	9.697	Adjusted for age ,LDL, blood Calcium,blood potassium, total cholesterol, alkaline phosphatase,Drinking Grouping and education level	6
Association of triglyceride-glucose index with the risk of prostate cancer: a retrospective study	Li, T.Q 2023-2	37953784	2023	China	Case-control study	2020-2022	In this study, 300 patients with prostate cancer diagnosed pathologically by prostate puncture biopsy in the First Affiliated Hospital of Xinjiang Medical University between 2020 and 2022 were retrospectively collected, and 3,000 healthy people without chronic or	Prostate cancer(ICD10-C61)	TyG Quartile 3 (8.373 to 8.854)	TyG Quartile 3 (8.373 to 8.854)	TyG Quartile 1 (<8.0)	190	34	NA	NA	71.00 (64.25,78.75)	NA	OR	6.918	2.275	21.043	Adjusted for age ,LDL, blood Calcium,blood potassium, total cholesterol, alkaline phosphatase,Drinking Grouping and education level	6

Association of triglyceride-glucose index with the risk of prostate cancer: a retrospective study	Li, T.Q 2023-3	37953784	2023	China	Case-control study	2020-2022	In this study, 300 patients with prostate cancer diagnosed pathologically by prostate puncture biopsy in the First Affiliated Hospital of Xinjiang Medical University between 2020 and 2022 were retrospectively collected, and 3,000 healthy people without chronic or	Prostate cancer(ICD10-C61)	TyG Quartile 4 (>=8.845)	TyG Quartile 4 (>=8.845)	TyG Quartile 1 (<8.0)	191	75	NA	NA	71.00 (64.25,78.75)	NA	OR	28.867	9.499	87.727	Adjusted for age ,LDL, blood Calcium,blood potassium, total cholesterol, alkaline phosphatase,Drinking Grouping and education level	6
The relationship between Triglyceride and glycose (TyG) index and the	Shi, H.M 2022-1	36184226	2022	China	Cross-sectional study	1999-2018	From the National Health and Nutrition Examination Survey (NHANSE, 1999 to 2018)	Gynaecologic and breast cancers	NA	Increase in TyG	NA	11466	586	0	11466	40.53±15.7	NA	OR	1.74	1.492	2.029	Adjusted for age, race, marial status ,BMI, HDL, LDL, Education, Age at menarche, Age at menopause	7
The relationship between Triglyceride and glycose (TyG) index and the	Shi, H.M 2022-2	36184226	2022	China	Cross-sectional study	1999-2018	From the National Health and Nutrition Examination Survey (NHANSE, 1999 to 2018)	Breast cancers	TyG Quartile 2(8.10 to 8.5)	TyG Quartile 2(8.10 to 8.5)	TyG Quartile 1(6.19 to 8.10)	2865	69	0	2865	47.8±17.8	NA	OR	1.693	1.14	2.52	Adjusted for age, race, marial status ,BMI, HDL, LDL, Education, Age at menarche, Age at menopause	7
The relationship between Triglyceride and glycose (TyG) index and the	Shi, H.M 2022-3	36184226	2022	China	Cross-sectional study	1999-2018	From the National Health and Nutrition Examination Survey (NHANSE, 1999 to 2018)	Breast cancers	TyG Quartile 3(8.50 to 9.00)	TyG Quartile 3(8.50 to 9.00)	TyG Quartile 1(6.19 to 8.10)	2867	98	0	2867	51.8±18.2	NA	OR	2.294	1.56	3.38	Adjusted for age, race, marial status ,BMI, HDL, LDL, Education, Age at menarche, Age at menopause	7
The relationship between Triglyceride and glycose (TyG) index and the	Shi, H.M 2022-4	36184226	2022	China	Cross-sectional study	1999-2018	From the National Health and Nutrition Examination Survey (NHANSE, 1999 to 2018)	Breast cancers	TyG Quartile 4(9.00 to 11.96)	TyG Quartile 4(9.00 to 11.96)	TyG Quartile 1(6.19 to 8.10)	2866	97	0	2866	54.5±17.1	NA	OR	2.25	1.5	3.37	Adjusted for age, race, marial status ,BMI, HDL, LDL, Education, Age at menarche, Age at menopause	7
The relationship between Triglyceride and glycose (TyG) index and the	Shi, H.M 2022-5	36184226	2022	China	Cross-sectional study	1999-2018	From the National Health and Nutrition Examination Survey (NHANSE, 1999 to 2018)	Ovarian cancers	TyG Quartile 3(8.50 to 9.00)	TyG Quartile 3(8.50 to 9.00)	TyG Quartile 1(6.19 to 8.10)	2867	16	0	2867	51.8±18.2	NA	OR	4.959	1.4	17.52	Adjusted for age, race, marial status ,BMI, HDL, LDL, Education, Age at menarche, Age at menopause	7
The relationship between Triglyceride and glycose (TyG) index and the	Shi, H.M 2022-6	36184226	2022	China	Cross-sectional study	1999-2018	From the National Health and Nutrition Examination Survey (NHANSE, 1999 to 2018)	Ovarian cancers	TyG Quartile 4(9.00 to 11.96)	TyG Quartile 4(9.00 to 11.96)	TyG Quartile 1(6.19 to 8.10)	2866	16	0	2866	54.5±17.1	NA	OR	3.734	1.01	13.87	Adjusted for age, race, marial status ,BMI, HDL, LDL, Education, Age at menarche, Age at menopause	7
The relationship between Triglyceride and glycose (TyG) index and the	Shi, H.M 2022-7	36184226	2022	China	Cross-sectional study	1999-2018	From the National Health and Nutrition Examination Survey (NHANSE, 1999 to 2018)	Uterine cancers	TyG Quartile 4(9.00 to 11.96)	TyG Quartile 4(9.00 to 11.96)	TyG Quartile 1(6.19 to 8.10)	2866	38	0	2866	54.5±17.1	NA	OR	2.424	1.14	5.16	Adjusted for age, race, marial status ,BMI, HDL, LDL, Education, Age at menarche, Age at menopause	7
Triglyceride-glucose index is a predictor of the risk of prostate cancer: a retrospective study based on a transprostatic aspiration	Zhou, Y.J 2024	38260162	2024	China	Case control study	2017-2021	The examination results of 316 patients who underwent prostate biopsy punctures from March 2017 to July 2021 in the First Affiliated Hospital of Xinjiang Medical University were retrospectively collected and patients	Prostate cancer	TyG Quartile 4(>9.241)	TyG Quartile 4(>9.241)	TyG Quartile 1(<8.389)	78	44	78	0	NA	NA	OR	2.854	1.2	6.79	Adjusted for age, initial PSA, smoking history, alcohol consumption, family history of cancer, BMI, TC, and LDL	6
Association of the triglyceride-glucose index with the occurrence and recurrence of colorectal adenomas:	Li, J.Y 2024-1	38395868	2024	China	Case control study	2020-2022	We retrospectively analyzed 16537 participants who underwent complete colonoscopy at a hospital in China from January 2020 to September 2022.	Colorectal adenoma	NA	Per 1 unit Increase in TyG	NA	3061	1596	NA	NA	NA	NA	OR	1.225	1.027	1.46	Adjusted for sex, age, systolic blood pressure, history of cancer, hypertension, hyperglycemia, dyslipidemia, history of smoking, family history of colorectal cancer, and high-density	7
Association of the triglyceride-glucose index with the occurrence and recurrence of colorectal adenomas:	Li, J.Y 2024-2	38395868	2024	China	Case control study	2020-2022	We retrospectively analyzed 16537 participants who underwent complete colonoscopy at a hospital in China from January 2020 to September 2022.	Colorectal adenoma	TyG Quartile 2(8.13 to 8.5)	TyG Quartile 2(8.13 to 8.5)	TyG Quartile 1(<8.13)	761	410	NA	NA	NA	NA	OR	1.324	1.02	2	Adjusted for sex, age, systolic blood pressure, history of cancer, hypertension, hyperglycemia, dyslipidemia, history of smoking, family history of colorectal cancer, and high-density	7
Association of the triglyceride-glucose index with the occurrence and recurrence of colorectal adenomas:	Li, J.Y 2024-3	38395868	2024	China	Case control study	2020-2022	We retrospectively analyzed 16537 participants who underwent complete colonoscopy at a hospital in China from January 2020 to September 2022.	Colorectal adenoma	TyG Quartile 3(8.51 to 8.94)	TyG Quartile 3(8.51 to 8.94)	TyG Quartile 1(<8.13)	754	413	NA	NA	NA	NA	OR	1.349	1.03	1.765	Adjusted for sex, age, systolic blood pressure, history of cancer, hypertension, hyperglycemia, dyslipidemia, history of smoking, family history of colorectal cancer, and high-density	7
Association of the triglyceride-glucose index with the occurrence and recurrence of colorectal adenomas:	Li, J.Y 2024-4	38395868	2024	China	Case control study	2020-2022	We retrospectively analyzed 16537 participants who underwent complete colonoscopy at a hospital in China from January 2020 to September 2022.	Colorectal adenoma	TyG Quartile 4(>8.94)	TyG Quartile 4(>8.94)	TyG Quartile 1(<8.13)	772	427	NA	NA	NA	NA	OR	1.445	1.055	1.98	Adjusted for sex, age, systolic blood pressure, history of cancer, hypertension, hyperglycemia, dyslipidemia, history of smoking, family history of colorectal cancer, and high-density	7
Association between triglyceride glucose index and breast cancer in 142,184 Chinese adults: findings from the REACTION study	Wu, X.Y 2024-1	38904041	2024	China	Cross-sectional study	2011-2012	This cross-sectional study included 142,184 women from the REACTION (Risk Evaluation of Cancers in Chinese Diabetic Individuals: A Longitudinal) Study, which recruited adults aged 40 years or older from 25 centers across mainland China between 2011 and 2012.	Breast cancer	NA	Per 1 SD increase	NA	142184	809	0	142184	56.37 ± 9.30	NA	OR	1.29	1.08	1.53	Adjusted for age, BMI, smoking status (current smoker or not), drinking status (current drinker or not), physical activity (moderate to vigorous or none to mild), family history ofbreast cancer, healthy diet (yes or no), 2h-PG, HbA1c and HDL-C, age at menarche, menopausal status (yes or no), and number of childbirths	9
Association between triglyceride glucose index and breast cancer in 142,184 Chinese adults: findings from the REACTION study	Wu, X.Y 2024-2	38904041	2024	China	Cross-sectional study	2011-2012	This cross-sectional study included 142,184 women from the REACTION (Risk Evaluation of Cancers in Chinese Diabetic Individuals: A Longitudinal) Study, which recruited adults aged 40 years or older from 25 centers across mainland China between 2011 and 2012.	Breast cancer	TyG Quartile 2(8.28 to 8.63)	TyG Quartile 2(8.28 to 8.63)	TyG Quartile 1(<8.28)	35544	218	0	35544	56.15 ± 9.29	NA	OR	1.51	1.14	2	Adjusted for age, BMI, smoking status (current smoker or not), drinking status (current drinker or not), physical activity (moderate to vigorous or none to mild), family history ofbreast cancer, healthy diet (yes or no), 2h-PG, HbA1c and HDL-C, age at menarche, menopausal status (yes or no), and number of childbirths	9
Association between triglyceride glucose index and breast cancer in 142,184 Chinese adults: findings from the REACTION study	Wu, X.Y 2024-3	38904041	2024	China	Cross-sectional study	2011-2012	This cross-sectional study included 142,184 women from the REACTION (Risk Evaluation of Cancers in Chinese Diabetic Individuals: A Longitudinal) Study, which recruited adults aged 40 years or older from 25 centers across mainland China between 2011 and 2012.	Breast cancer	TyG Quartile 3(8.64 to 9.02)	TyG Quartile 3(8.64 to 9.02)	TyG Quartile 1(<8.28)	35548	198	0	35548	57.62 ± 9.10	NA	OR	1.44	1.08	1.93	Adjusted for age, BMI, smoking status (current smoker or not), drinking status (current drinker or not), physical activity (moderate to vigorous or none to mild), family history ofbreast cancer, healthy diet (yes or no), 2h-PG, HbA2c and HDL-C, age at menarche, menopausal status (yes or no), and number of childbirths	9
Association between triglyceride glucose index and breast cancer in 142,184 Chinese adults: findings from the REACTION study	Wu, X.Y 2024-4	38904041	2024	China	Cross-sectional study	2011-2012	This cross-sectional study included 142,184 women from the REACTION (Risk Evaluation of Cancers in Chinese Diabetic Individuals: A Longitudinal) Study, which recruited adults aged 40 years or older from 25 centers across mainland China between 2011 and 2012.	Breast cancer	TyG Quartile 4(>9.02)	TyG Quartile 4(>9.02)	TyG Quartile 1(<8.28)	35546	255	0	35546	58.38 ± 8.72	NA	OR	1.61	1.19	2.17	Adjusted for age, BMI, smoking status (current smoker or not), drinking status (current drinker or not), physical activity (moderate to vigorous or none to mild), family history ofbreast cancer, healthy diet (yes or no), 2h-PG, HbA3c and HDL-C, age at menarche, menopausal status (yes or no), and number of childbirths	8
Triglyceride-glucose index is a risk factor for breast cancer in China: a cross-sectional study	Zhang, J.H 2024	38279158	2024	China	Cross-sectional study	2020-2021	A retro analysis was made with 3,097 sufferers under-going breast disease admitted to the Department of Surgery, Women’s Hospital, Zhejiang University School of Medicine, from March 2020 to	Breast cancer	TyG Quartile 4(8.63 to 10.89)	TyG Quartile 4(8.63 to 10.89)	TyG Quartile 1(6.88 to 7.87)	639	170	0	639	42.17 ± 11.78	NA	OR	1.43	1.01	2.02	Adjusted for age, BMI, smoking status, drinking status, hypertension, family history of malignancy, age at menarche, and hormonal contraception	8
Relationship between obesity indexes and triglyceride glucose index with gastrointestinal cancer among the US	Zha, B.W 2024-1	38818028	2024	China	Cross-sectional study	1999-2018	From National Health and Nutrition Examination Survey (NHANSE, 1999 to 2018)	Gastrointestinal cancers	TyG Quartile 4	TyG Quartile 4	TyG Quartile 1	NA	NA	NA	NA	NA	NA	OR	2.082	1.016	4.269	Adjusted for gender, age, education level, race, smoking and drinking	9
Relationship between obesity indexes and triglyceride glucose index with gastrointestinal cancer among the US	Zha, B.W 2024-2	38818028	2024	China	Cross-sectional study	1999-2018	From National Health and Nutrition Examination Survey (NHANSE, 1999 to 2018)	Gastrointestinal cancers	TyG Quartile 4;Female	TyG Quartile 4	TyG Quartile 1	NA	NA	NA	NA	NA	NA	OR	4.808	1.695	13.643	Adjusted for gender, age, education level, race, smoking and drinking	9
Relationship between obesity indexes and triglyceride glucose index with gastrointestinal cancer	Zha, B.W 2024-3	38818028	2024	China	Cross-sectional study	1999-2018	From National Health and Nutrition Examination Survey (NHANSE, 1999 to 2018)	Gastrointestinal cancers	TyG Quartile 4;Age >60	TyG Quartile 4	TyG Quartile 1	NA	NA	NA	NA	NA	NA	OR	2.879	1.373	6.036	Adjusted for gender, age, education level, race, smoking and drinking	9
Relationship between obesity indexes and triglyceride glucose index with gastrointestinal cancer	Zha, B.W 2024-4	38818028	2024	China	Cross-sectional study	1999-2018	From National Health and Nutrition Examination Survey (NHANSE, 1999 to 2018)	Gastrointestinal cancers	TyG Quartile 2;No-Smoker	TyG Quartile 2	TyG Quartile 1	NA	NA	NA	NA	NA	NA	OR	4.548	1.283	16.117	Adjusted for gender, age, education level, race, smoking and drinking	9
Relationship between obesity indexes and triglyceride glucose index with gastrointestinal cancer	Zha, B.W 2024-5	38818028	2024	China	Cross-sectional study	1999-2018	From National Health and Nutrition Examination Survey (NHANSE, 1999 to 2018)	Gastrointestinal cancers	TyG Quartile 3;No-Smoker	TyG Quartile 3	TyG Quartile 1	NA	NA	NA	NA	NA	NA	OR	3.897	1.105	13.745	Adjusted for gender, age, education level, race, smoking and drinking	9
Relationship between obesity indexes and triglyceride glucose index with gastrointestinal cancer among the US	Zha, B.W 2024-6	38818028	2024	China	Cross-sectional study	1999-2018	From National Health and Nutrition Examination Survey (NHANSE, 1999 to 2018)	Gastrointestinal cancers	TyG Quartile 4;No-Smoker	TyG Quartile 4	TyG Quartile 1	NA	NA	NA	NA	NA	NA	OR	4.825	1.417	16.423	Adjusted for gender, age, education level, race, smoking and drinking	9
Relationship between obesity indexes and triglyceride glucose index with gastrointestinal cancer	Zha, B.W 2024-7	38818028	2024	China	Cross-sectional study	1999-2018	From National Health and Nutrition Examination Survey (NHANSE, 1999 to 2018)	Gastrointestinal cancers	TyG Quartile 4;No-Drinker	TyG Quartile 4	TyG Quartile 1	NA	NA	NA	NA	NA	NA	OR	5.653	1.403	22.774	Adjusted for gender, age, education level, race, smoking and drinking	9
The Association Between Triglyceride-Glucose Index as a Marker of	Panigoro, S 2021-1	34707572	2021	Indonesia	Case-control study	2019-2019	This is a multicenter case-control study conducted in six public referral hospitals in Indonesia.	Breast cancer	TyG Quartile 2(8.13 to 8.47)	TyG Quartile 2(8.13 to 8.47)	TyG Quartile 1(7.09 to 8.12)	108	52	0	108	NA	NA	OR	1.39	1.12	1.73	NA	6
The Association Between Triglyceride-Glucose Index as a Marker of	Panigoro, S 2021-2	34707572	2021	Indonesia	Case-control study	2019-2019	This is a multicenter case-control study conducted in six public referral hospitals in Indonesia.	Breast cancer	TyG Quartile 3(8.48 to 8.86)	TyG Quartile 3(8.48 to 8.86)	TyG Quartile 1(7.09 to 8.12)	108	57	0	108	NA	NA	OR	1.53	1.21	1.93	NA	6
The Association Between Triglyceride-Glucose Index as a Marker of	Panigoro, S 2021-3	34707572	2021	Indonesia	Case-control study	2019-2019	This is a multicenter case-control study conducted in six public referral hospitals in Indonesia.	Breast cancer	TyG Quartile 2(8.87 to 10.90)	TyG Quartile 2(8.87 to 10.90)	TyG Quartile 1(7.09 to 8.12)	107	75	0	107	NA	NA	OR	2.42	1.77	3.31	NA	6
The Association Between Triglyceride-Glucose Index as a Marker of	Panigoro, S 2021-4	34707572	2021	Indonesia	Case-control study	2019-2019	This is a multicenter case-control study conducted in six public referral hospitals in Indonesia.	Breast cancer	TyG index >8.87	TyG index >8.87	TyG index <8.87	424	212	0	424	NA	NA	OR	2.93	1.72	4.98	Adjusted for age,early menarche,family history of malignancy,smoking,use of contraception,alcohol	6

Association Between Triglyceride Glucose Index and Non-Small Cell Lung Cancer Risk in Chinese Population	Yan, X 2021	33777737	2021	China	Case-control study	2016-2018	We retrospectively collected 791 newly diagnosed and pathologically confirmed NSCLC patients between 2016 and 2018 at the Department ofRespiration ofNanjing Drum Tower Hospital.	Non-small cell lung cancer	NA	Per 1 unit Increase in TyG	NA	1578	791	678	900	NA	NA	OR	3.651	2.461	5.417	Adjustment for age, sex, smoking, BMI, hypertension, WBCC, Neutrophil count, TC, LDL-C, HDL-C and uric acid.	6
The relationship between papillary thyroid cancer and triglyceride/glucose index, which [illegible]	Alkurt, E.G 2022	36111913	2022	Turkey	Case-control study	2013-2021	The patients who applied to the Department of General Surgery who under-went bilateral total thyroidectomy between 2013and 2021 were scanned and a total of 1,517 pa-tients were identified.	Papillary thyroid cancer	TyG index >6.252	TyG index >6.252	TyG index <6.252	382	254	97	285	NA	NA	OR	2.147	1.387	3.323	NA	7
Triglyceride glucose index and Atherogenic index of plasma for predicting colorectal neoplasms in patients without cardiovascular diseases	Han, M.Z 2022-1	36452491	2022	China	Case-control study	2016-2019	This is a single-center retrospective analysis of 2835 were admitted to Beijing Friendship Hospital. They underwent a colonoscopy during the period January 1, 2016 to December 31, 2019.	Colorectal neoplasms	TyG Quartile 4	TyG Quartile 4	TyG Quartile 1	NA	NA	NA	NA	57.18 ± 11.26	NA	OR	1.35	1.02	1.77	Adjusted for age, sex, family history and FOBT(fecal occult blood test)	8
Triglyceride glucose index and Atherogenic index of plasma for predicting colorectal neoplasms in patients without cardiovascular diseases	Han, M.Z 2022-2	36452491	2022	China	Case-control study	2016-2019	This is a single-center retrospective analysis of 2835 were admitted to Beijing Friendship Hospital. They underwent a colonoscopy during the period January 1, 2016 to December 31, 2019.	Colorectal neoplasms	NA	Per 1unit Increase in TyG	NA	2409	1462	1355	1054	57.18 ± 11.26	NA	OR	1.19	1.01	1.4	Adjusted for age, sex, family history and FOBT(fecal occult blood test)	8
