# Supplementary material for: The Associations Between the TyG Index and the Risk of Cancer—A Systematic Review and Meta‐Analysis
Source: Cancer Med. 2025 Oct 2;14(19):e71232. doi: 10.1002/cam4.71232 (PMC12489462; doi:10.1002/cam4.71232)
Supplement: Supplementary file 4 — Appendix S3: Quality assessment of included studies. This appendix details the methodological quality for each included study, generated using the [Newcastle‐Ottawa Scale (NOS) and the Agency for Healthcare Research and Quality (AHRQ)] tool. It includes the rationale for each judgment and a summary of scores. [file CAM4-14-e71232-s001.pdf]

Title	Selection				Comparability		Exposure/Outcome			Total NOS scores		
	Definition of Cohort	Definition of Control	Representativity	Follow-time	Baseline comparability	Confounding factor contro	Exposure Measurement	Measurement of endings	Follow-up completeness			
**Triglyceride–glucose index (TyG index) is a predictor of incident colorectal cancer: a population-based longitudinal study**	0 (No clear exclusion criteria for inclusion)	0 (No clear exclusion criteria for inclusion)	0 (Population of medical examinations from only one hospital)	1	1	1	1	0（No clear ICD code）	1	5		
**The triglyceride-glucose index as a measure of insulin resistance and risk of obesity-related cancers**	1	1	1	1	1	1	1	1	1	9		
**Association between triglyceride-glucose index and gastric carcinogenesis: a health checkup cohort study**	1	1	0 (Endoscopy population from only one hospital)	1	1	1	1	0（No clear ICD code）	1	7		
**Association between the TyG index and TG/ HDL-C ratio as insulin resistance markers and the risk of colorectal cancer**	1	1	0 (Active and retired employees of a group only)	1	0 (Significant gender bias)	1 (A stratified analysis was conducted)	1	1	1	7		
**Association between Surrogate Markers of Insulin Resistance and the Incidence of Colorectal Cancer in Korea: A Nationwide Population-Based Study**	1	1	1	1	1	1	1	0（No clear ICD code）	1	8		
**Triglyceride-glucose index (TyG index) and endometrial carcinoma risk: A retrospective cohort study**	1	1	0 (Population of medical examinations from only one hospital)	0 (Unclear definition)	1	1	1	0（No clear ICD code）	0 (two years in review,Unclear definition)	5		
**Association between four insulin resistance surrogates and the risk of esophageal cancer: a prospective cohort study using the UK Biobank**	1	1	1	1	1	1	1	0（No clear ICD code）	0 (No follow-up deadline given)	7		
**Triglyceride-Glucose Index, Modifiable Lifestyle, and Risk of Colorectal Cancer: A Prospective Analysis of the Korean Genome and Epidemiology Study**	1	1	1	1	0 (Significant gender bias)	1 (A stratified analysis was conducted)	1	1	1	8		
**Triglyceride-Glucose Index Is Not Associated With Lung Cancer Risk: A Prospective Cohort Study in the UK Biobank**	1	1	1	1	1	1	1	1	1	9		
**Title**	Selection				Comparability		Exposure/Outcome			Total NOS scores		
	Definition of Case	Representativeness of Case	Selection of Control	Definition of Control	the most important factor	additional factor	Exposure Measurement	Same method of ascertainment for cases and controls	Non-Response rate			
**Association of triglyceride-glucose index with the risk of prostate cancer: a retrospective study**	1	1	0 (hospital controls)	1	0（Significant age bias)	1	1	1	0（non respondents described）	6		
**Triglyceride-glucose index is a predictor of the risk of prostate cancer: a retrospective study based on a transprostatic aspiration biopsy population**	1	1	0 (hospital controls diagnosed with benign prostatic hyperplasia)	1	0（Significant age bias)	1	1	1	0（non respondents described）	6		
**The relationship between Triglyceride and glycose (TyG) index and the risk of gynaecologic and breast cancers**	0 (based on self reports	1	1	1	1	1	1	1	0（non respondents described）	7		
**Association of the triglyceride-glucose index with the occurrence and recurrence of colorectal adenomas: a retrospective study from China**	1	1	0（Population underwent complete colonoscopy from only one hospital)	1	1	1	1	1	0（non respondents described）	7		
**The Association Between Triglyceride-Glucose Index as a Marker of Insulin Resistance and the Risk of Breast Cancer**	1	1	0(Controls were recruited from corresponding hospitals)	1	0（Significant age bias)	1	1	1	0（non respondents described）	6		
**Association Between Triglyceride Glucose Index and Non-Small Cell Lung Cancer Risk in Chinese Population**	1	1	0(randomly selected following examination in the clinic and were classified as the control group)	1	0（Significant age bias)	1	1	1	0（non respondents described）	6		
**The relationship between papillary thyroid cancer and triglyceride/glucose index, which is an indicator of insulin resistance**	1	1	0 (hospital controls)	1	1	1	1	1	0（non respondents described）	7		
**Triglyceride glucose index and Atherogenic index of plasma for predicting colorectal neoplasms in patients without cardiovascular diseases**	1	1	0 (hospital controls)	1	1	1	1	1	1	8		
**Title**	1. Define the source of information (survey, record review).	2. List inclusion and exclusion criteria for exposed and unexposed subjects (cases and controls) or refer to previous publications.	3. Indicate time period used for identifying patients.	4. Indicate whether or not subjects were consecutive if not population-based.	5. Indicate if evaluators of subjective components of study were masked to other aspects of the participants.	6. Describe any assessments undertaken for quality assurance purposes (e.g.,test/retest of primary outcome measurements).	7. Explain any patient exclusions from analysis.	8. Describe how confounding was assessed and/or controlled.	9. If applicable, explain how missing data were handled in the analysis.	10. Summarize patient response rates and completeness of data collection.	11. Clarify what follow-up, if any, was expected and the percentage of patients for which incomplete data or follow-up was obtained.	Total

**Association between triglyceride glucose index and breast cancer in 142,184 Chinese adults: findings from the REACTION study**	1	1	1	1	1	1	1	1	0	1	0	9
**Triglyceride-glucose index is a risk factor for breast cancer in China: a cross-sectional study**	1	1	1	1	1	1	1	1	0	0	0	8
**Relationship between obesity indexes and triglyceride glucose index with gastrointestinal cancer among the US population**	1	1	1	1	1	1	1	1	0	1	0	9
