# Supplementary material for: The Associations Between the TyG Index and the Risk of Cancer—A Systematic Review and Meta‐Analysis
Source: Cancer Med. 2025 Oct 2;14(19):e71232. doi: 10.1002/cam4.71232 (PMC12489462; doi:10.1002/cam4.71232)
Supplement: Supplementary file 5 — Appendix S4: Subgroup analyses of the association between the triglyceride‐glucose (TyG) index and cancer risk. (A) Subgroup analyses for the association between the TyG index as a continuous variable and cancer risk. (B) Subgroup analysis for the association between high versus low levels of the TyG index (categorical variable) and cancer risk. [file CAM4-14-e71232-s002.pdf]

Subgroup Analysis for the association between Categorical TyG index and Cancer

Disease	No.of Studies	Sample Size	I^2(%)	Pooled OR (95%CI)	*P* for Interaction
**(A)**					
**Digestive system**	7	1363503	59.077	1.132(1.081-1.186)	
**Study Design**					0.497
Cohort	6	1361094	59.828	1.122(1.074-1.172)	
Case-control	1	2409	NA	1.190(1.011-1.401)	
**Geographic Background**					0.006
Asia	5	464132	0	1.200(1.136-1.267)	
Europe	2	899371	62.248	1.083(1.032-1.136)	
**Source of Participants**					0.574
Employee	1	93659	NA	1.190(1.053-1.344)	
Health check-up	3	271673	0	1.145(1.055-1.243)	
Multicenter	3	998171	66.301	1.114(1.059-1.171)	
**Sex**					0.85
Female	3	850614	0	1.195(1.114-1.282)	
Male	3	850614	58.082	1.181(1.070-1.304)	
**(B)**					
**Digestive system**	7	1342202	70.576	1.218(1.133-1.308)	
**Study Design**					0.222
Case-control	1	2409	NA	1.350(1.025-1.778)	
Cohort	5	1318382	74.378	1.188(1.098-1.285)	
Cross-sectional	1	21411	NA	2.082(1.016-4.268)	
**Geographic Background**					0.095
America	1	21411	NA	2.082(1.016-4.268)	
Asia	4	421420	69.78	1.261(1.139-1.396)	
Europe	2	899371	77.657	1.108(0.978-1.256)	
**Source of Participants**					
Employee	1	93659	NA	1.433(1.205-1.704)	0.083
Health check-up	3	327761	69.038	1.209(1.089-1.343)	
Multicenter	3	920782	76.714	1.125(0.992-1.274)	
**Sex**					0.365
Female	2	899371	53.554	1.746(1.221-2.498)	
Male	1	93659	NA	1.450(1.209-1.740)	
**Urogenital system**	4	523020	80.988	2.038(1.534-2.709)	
**Study Design**					0.00
Case-control	2	1083	74.625	6.351(2.242-17.990)	
Cohort	1	510471	NA	1.284(1.168-1.412)	
Cross-sectional	1	11466	NA	3.070(1.717-5.488)	
**Geographic Background**					0.00
America	1	11466	NA	3.070(1.717-5.488)	
Asia	2	1083	74.625	6.351(2.242-17.990)	
Europe	1	510471	NA	1.284(1.168-1.412)	
**Source of Participants**					0.004
Health check-up	2	1083	74.625	6.351(2.242-17.990)	
Multicenter	2	521937	40.524	1.346(1.170-1.548)	
**Breast Cancer**	4	156662	41.852	1.635(1.488-1.796)	
**Study Design**					0.811
Case-control	1	424	NA	1.614(1.400-1.860)	
Cross-sectional	3	156238	13.924	1.652(1.456-1.873)	
**Geographic Background**					0.029
America	1	11466	NA	2.061(1.640-2.590)	
Asia	3	145196	35.464	1.559(1.406-1.729)	
**Source of Participants**					0.432
Health check-up	1	2588	NA	1.430(1.011-2.022)	
Multicenter	3	154074	46.161	1.652(1.498-1.822)	

(A) Subgroup analysis of the association between the continuous TyG index and cancer.

(B) Subgroup analysis of cancer risk with high levels of The yG index compared to low levels of the TyG index.
